# Biocatalytic Synthesis and Polymerization via ROMP of New Biobased Phenolic Monomers: A Greener Process toward Sustainable Antioxidant Polymers

**DOI:** 10.3389/fchem.2017.00126

**Published:** 2017-12-22

**Authors:** Florian Diot-Néant, Loïs Migeot, Louis Hollande, Felix A. Reano, Sandra Domenek, Florent Allais

**Affiliations:** ^1^Chaire ABI, AgroParisTech, CEBB, Pomacle, France; ^2^The George and Josephine Butler Laboratory for Polymer Research, Department of Chemistry, University of Florida, Gainesville, FL, United States; ^3^UMR GENIAL, AgroParisTech, Institut National De la Recherche Agronomique, Université Paris-Saclay, Massy, France; ^4^UMR 782 GMPA, AgroParisTech, Institut National de la Recherche Agronomique, Université Paris-Saclay, Thiverval-Grignon, France

**Keywords:** antioxidants, ROMP, lipase, CAL-B, norbornene, ferulic acid, sinapic acid, DPPH

## Abstract

Antioxidant norbornene-based monomers bearing biobased sterically hindered phenols (SHP)—**NDF** (norbornene dihydroferulate) and **NDS** (norbornene dihydrosinapate)—have been successfully prepared through biocatalysis from naturally occurring ferulic and sinapic acids, respectively, in presence of *Candida antarctica Lipase B* (Cal-B). The ring opening metathesis polymerization (ROMP) of these monomers was investigated according to ruthenium catalyst type (GI) vs. (HGII) and monomer to catalyst molar ratio ([M]/[C]). The co-polymerization of antioxidant functionalized monomer (**NDF** or **NDS**) and non-active norbornene (**N**) has also been performed in order to adjust the number of SHP groups present per weight unit and tune the antioxidant activity of the copolymers. The polydispersity of the resulting copolymers was readily improved by a simple acetone wash to provide antioxidant polymers with well-defined structures. After hydrogenation with *p*-toluenesulfonylhydrazine (*p*-TSH), the radical scavenging ability of the resulting saturated polymers was evaluated using α,α-diphenyl-β-picrylhydrazyl (DPPH) analysis. Results demonstrated that polymers bearing sinapic acid SHP exhibited higher antiradical activity than the polymer bearing ferulic acid SHP. In addition it was also shown that only a small SHP content was needed in the copolymers to exhibit a potent antioxidant activity.

## Introduction

The fundamental principle for the stabilization of polymeric materials consists in preserving the initial aesthetic and mechanical properties of a polymer during processing, storage, and application. In fact, polymeric materials are commonly exposed to several external effects, such as heat, oxygen, radiation, residual metal catalyst, and mechanical stress which are well-known to promote undesirable deterioration of theses mechanical, physical and of course chemicals properties (Pospíšil, 1993; Zweifel, 1998). The addition of antioxidants in small amounts to polymers is a convenient and efficient way to drastically delay their deterioration. Many classes of antioxidant additives, often used in combination to achieve synergy, are commercialized and classified according to their mechanisms of action. Sterically hindered phenols (SHP) such as Irganox® 1010, Irganox® 1076, BHT, or Ethanox® 330, are by far the most commonly used class used in industry (Girois, 2013). These antioxidants belong to the group of free radical-scavengers and act like radical inhibitors (IH) based on the transfer of a hydrogen atom from the IH to the free radical species R^•^ or an oxidative associated product and results in less reactive products RH and I^•^.

Unfortunately, conventional antioxidants are not perfect and suffer from some serious limitations. Because of low molecular weight, antioxidants are easily subject to depart from the polymer matrix by physical loss such as migration, evaporation, leaching and extraction (Marcato et al., 2003; Dopico-García et al., 2007). This is a serious issue because food contamination by antioxidants or potentially by-products can negatively impact human health. In addition, the physical loss decreases the effective protective capabilities of antioxidant. Therefore, additives that are potentially able to migrate into food or ending up in environmental medium are strictly controlled by safety-regulations through composition limits in the plastic or migration limits (Code of Federal Regulations Title 21, 2013).

The most common strategy used to limit the physical loss of stabilizers is increasing their molecular weight to minimize their mobility (Kasza et al., 2015). Two general methods to synthesize these larger antioxidant polymers have been reported: chemical modification of pre-formed polymers, or polymerization of antioxidant monomers. The first approach involves the post-functionalization of reactive groups of natural (Arefjev et al., 1999; Sousa et al., 2009) or synthetic (Kim and Oh, 2004) polymer chains with sterically hindered phenol groups (SHP) with the aim to improve their antioxidant activity. This method is quite efficient and easily accessible, but may trigger undesirable side reactions, especially regarding the difficulty to obtain full distribution of stabilizers at the reactive sites of the matrix. In addition, part of SHP groups is consumed or subject to homopolymerization during grafting reactions, rending difficult the preparation of polymers with a controllable structure. The second approach consists in the oligomerization of antioxidant-functionalized monomers. In this case, the monomer design is the key point for adjusting final performances of functionalized polymers. Moreover, depending on the type of polymerization and catalyst used, this approach provides more control and flexibility of the resulting structure of the antioxidant polymers. Many elegant and efficient methods have been developed to design antioxidant monomers (e.g., vinyl, acryl, styrenic) which can be useful in conventional polymerization techniques (Xue et al., 2007). Unfortunately, the major drawbacks of these reactions are the highly carcinogenic by-products formed or the often-hazardous reagents used in these synthetic pathways (e.g., dichloromethyl (Campbell et al., 2001), or alkenylchloride).

**Figure 1:**
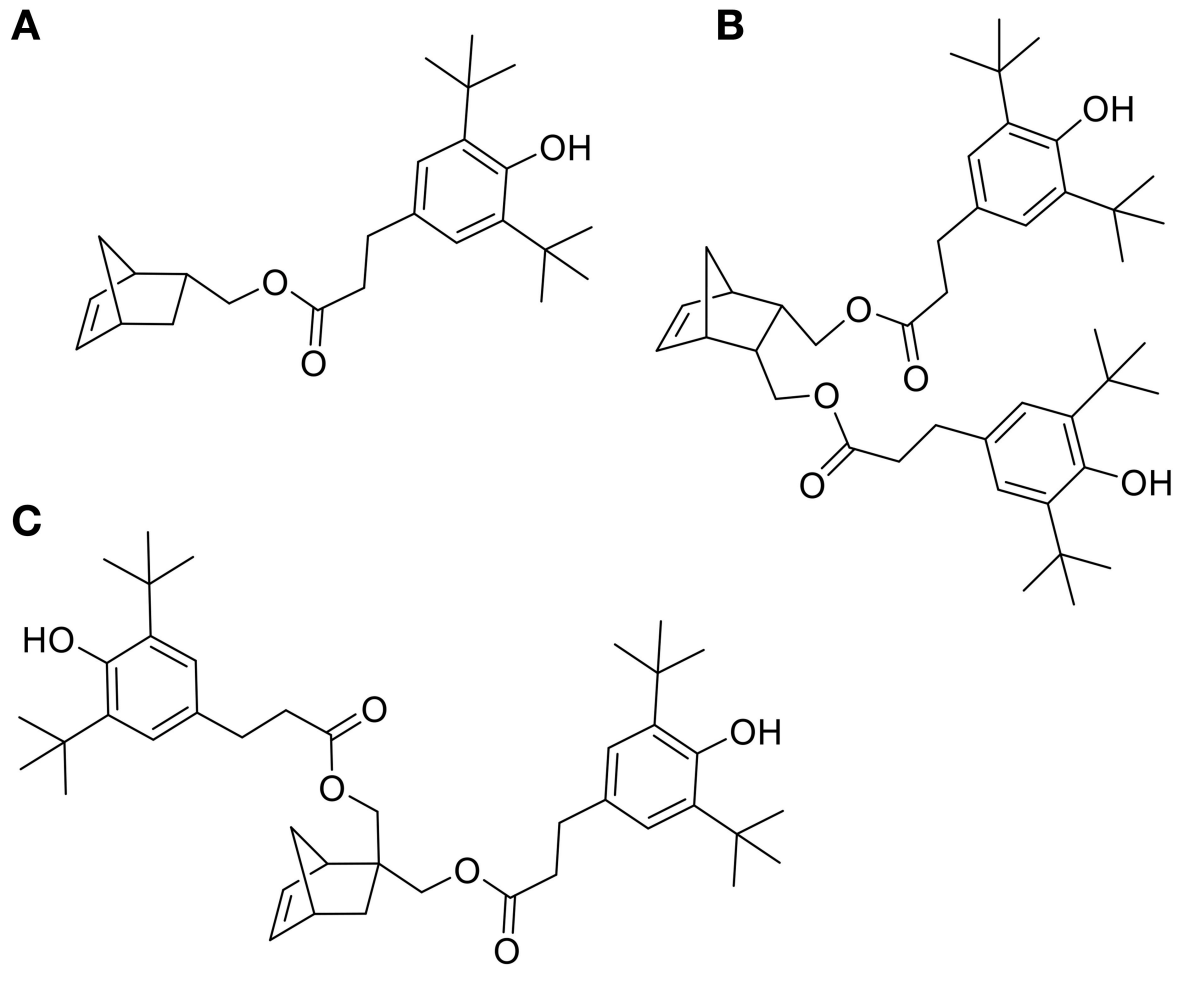
Monomers **(A–C)** used in ROMP by Xue.

ROMP is an attractive and versatile tool for preparing functional and linear polymers or copolymers. Grubbs et al. reported a family of well-defined ruthenium catalysts for ROMP (Schwab et al., 1996). Although ruthenium catalysts show activities much lower that of the early transition metals catalysts, they provide a clearly better balance between activity and significant tolerance toward functional groups (Nguyen and Grubbs, 1993; Dias et al., 1997; Scherman et al., 2002). Xue and coworkers reported on the synthesis and ROMP of a family of norbornene monomers bearing one or two phenolic antioxidant moieties (Figures 1A–C). However, these compounds and their synthetic procedure have major drawbacks, indeed not only the SHP used - 3,5-di-*tert*-butyl-4-hydroxylphenoxyacetyl chloride - is fossil-based but the synthetic process also involves the use of pyridine, a recognized toxic chemical (Xue et al., 2007, 2008).

In the present work, we report a new and greener pathway to biobased antioxidant phenolic polymers through the chemo-enzymatic synthesis of new hindered phenol-functionalized norbornene derivatives from naturally occurring ferulic and sinapic acids using *Candida antarctica* Lipase B (Cal-B) and their homo/copolymerization using ROMP. Ferulic acid, naturally present at relatively high concentrations in the cell walls of several plants, is known for acting as a free radical scavenger due to its hydrogen donating ability. Many researchers have engaged in high scale production of this acid using techniques like extraction from agricultural wastes (Tilay et al., 2008), enzymatic fractionation (Dupoiron et al., 2017) or more recently by fermentation (Overhage et al., 2003). Ferulic acid can also be synthesized from vanillin using a Knovenagel condensation with malonic acid. Sinapic acid, offers an extra methoxy group at the *ortho*-position (electron donor) that further increases the antioxidant activity (Cuvelier et al., 1992). Similarly to ferulic acid, sinapic acid can be extracted directly from biomasses such as canola cake or mustard bran but can also be easily obtained from syringaldehyde by condensation with malonic acid as previously reported (Jaufurally et al., 2016).

## Experimental

### Materials

Syringaldehyde, malonic acid, ferulic acid, Pd/C, norbornene-endo-2,3-dicarboxylic anhydride and lithium aluminum hydride were purchased from Sigma-Aldrich. *Candida antarctica* Lipase B was purchased from Novozyme. Solvents were purchased from ThermoFisher Scientific and VWR. Deuterated chloroform (CDCl_3_) and acetone ((CD_3_)_2_CO) were purchased from Euriso-top. Column chromatographies were carried out with an automated flash chromatography (PuriFlash 4100, Interchim) and pre-packed INTERCHIM PF-30SI-HP (30 μm silica gel) columns using a gradient of cyclohexane and ethyl acetate for the elution. NMR analyses were recorded on a Bruker Fourier 300 (Supplementary Data Sheet 1). ^1^H NMR spectra of samples were recorded in CDCl_3_ at 300 MHz, chemical shifts were reported in parts per million (CDCl_3_, CHCl_3_ residual signal at δ = 7.26 ppm; Acetone-d_6_, acetone residual signal at δ = 2.05 ppm). ^13^C NMR spectra of samples were recorded at 75 MHz (CDCl_3_ signal at δ = 77.16 ppm; Acetone residual signal at δ = 29.84 and 206.26 ppm). HRMS were recorded by the PLANET platform at URCA on a Micromass GC-TOF.

Thermo-gravimetric analyses (TGA) were recorded on a Q500, from TA. About 10 mg of each sample was heated at 10 °C.min^−1^ from 30 to 500 °C under nitrogen flow (60 mL.min^−1^). Differential scanning calorimetry (DSC) thermograms were obtained using a DSC Q20 and Tzero Hermetic Lid pans from TA, under inert atmosphere (N_2_), with a calibration using indium, *n-*octadecane and *n-*octane standards. For each sample, about 10 mg were weighed in a pan which was then sealed and submitted to 3 heat/cool/heat cycles: heating from 30 °C to 200 °C at 10 °C.min^−1^, cooling from 200 °C to −50 °C at 20 °C.min^−1^. Glass transition temperatures (*Tg*) were determined at the inflexion value in the heat capacity jump.

Gel permeation chromatography (GPC) analyses were performed on an Agilent Infinity 1,260 equipped with four detectors (UV, λ = 280 nm, RI, LS, viscosimeter). About 3 mg of polymer of each sample was dissolved in 1 mL THF (stabilize with BHT). The column PL Gel MIXED-D (5 μm, 300 × 7.5 mm) was calibrated with polystyrene standards in THF flow (1 mL.min^−1^).

### Monomer synthesis

#### Synthesis of norbornene dihydroferulate (NDF)

In a round-bottom flask, *cis*-5-norbornene-2,3-endo-dimethanol (10.04 g, 69.22 mmol) are charged with ethyl (dihydro)ferulate (46.52 g, 207.66 mmol, 3 equiv) and CAL-B (*Candida antarctica* Lipase B, 5.66 g, 10% (w/w)). The mixture is heated at 75 °C under reduced pressure. After 3 days of reaction, the medium is solubilized in 250 mL of AcOEt and the supported enzyme is eliminated by filtration. After concentration, a yellow oil is obtained and purified by flash purification on silica gel (cyclohexane/AcOEt: 85/15, then 55/45) to provide a colorless oil (30.7 g, 87%). ^1^**H NMR (CDCl**_3_**, 300 MHz**, δ**):** 6.83–6.66 (m, 6H, H_5,8,9_), 6.10 (t, 2H, *J* = 1.8 Hz, ${\text{H}}_{1^{\text{′}}}$), 5.50 (s, 2H, H_11_), 3.86 (s, 6H, H_10_), 3.83–3.69 (m, 4H, ${\text{H}}_{5^{\text{′}}}$), 2.87 (t, 4H, *J* = 8.1 Hz, H_2_), 2.81 (m, 2H, ${\text{H}}_{2^{\text{′}}}$), 2.59 (t, 2H, *J* = 7.2 Hz, H_3_), 2.46 (m, 2H, ${\text{H}}_{4^{\text{′}}}$), 1.47 (app.dd, 1H, *J* = 8.4 Hz, ${\text{H}}_{3^{\text{′}}}$), 1.27 (app.dd, 1H, *J* = 8.4 Hz, ${\text{H}}_{3^{\text{′}}}$). ^13^**C NMR (CDCl**_3_**, 75 MHz**, δ**):** 172.9 (C_1_), 146.5 (C_6_), 144.2 (C_7_), 135.5 (${\text{C}}_{1^{\text{′}}}$), 132.5 (C_4_), 120.9 (C_5_), 114.4 (C_8_), 111.1 (C_6_), 64.6 (${\text{C}}_{5^{\text{′}}}$), 56.0 (C_10_), 49.1 (${\text{C}}_{3^{\text{′}}}$), 45.6 (${\text{C}}_{2^{\text{′}}}$), 40.7 (${\text{C}}_{4^{\text{′}}}$), 36.4 (C_2_), 30.9 (C_3_). **FT-IR (neat)**: ν_max_ 3443 (ArOH), 1723 (C = O), 1603–1450 (C = C_Ar_). **UV (nm)**: λ_max_: 215. **HRMS (TOF MS, ES**+**)**: *m/z* calcd for C_29_H_34_O_8_Na: 533.2151; found: 533.2161


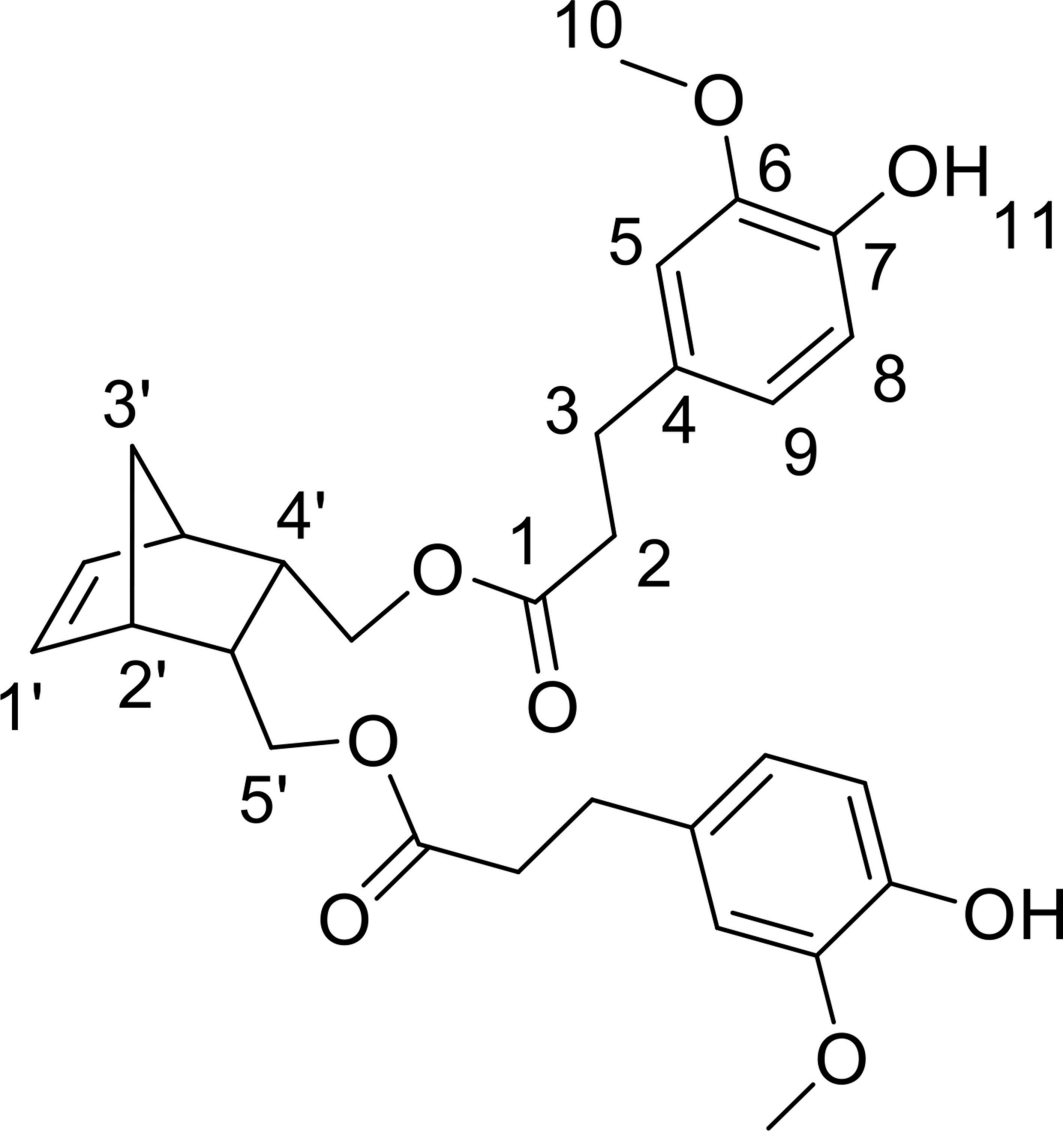


#### Synthesis of norbornene dihydrosinapate (NDS)

In a round-bottom flask, *cis*-5-norbornene-2,3-endo-dimethanol (2.01 g, 13.03 mmol) are charged with of ethyl (dihydro)sinapate (9.89 g, 39.1 mmol, 3 equiv) and CAL-B (*Candida antarctica* Lipase B, 1.19 g (10% w/w)). The mixture is heated at 75 °C under reduced pressure. After 12 days of reaction, the medium is solubilized in 100 mL of AcOEt and the supported enzyme is eliminated by filtration. After concentration, a yellow oil is obtained and purified by flash purification on silica gel (cyclohexane/AcOEt: 85/15, then 55/45) to provide a colorless oil (4.462 g, 60%). ^1^**H NMR (CDCl**_3_**, 300 MHz**, δ**)**: 6.43 (s, 4H, H_5_), 6.11 (t, 2H, *J* = 1.8 Hz, ${\text{H}}_{1^{\text{′}}}$), 5.39 (s, 2H, H_9_), 3.87 (s, 12H, H_8_), 3.82–3.70 (m, 4H, ${\text{H}}_{5^{\text{′}}}$), 2.88 (t, 4H, *J* = 8.1 Hz, H_2_), 2.83 (m, 2H, ${\text{H}}_{2^{\text{′}}}$), 2.61 (t, 4H, *J* = 7.2 Hz, H_3_), 2.45 (m, 2H, ${\text{H}}_{4^{\text{′}}}$), 1.50 (app.dd, 1H, *J* = 8.4 Hz, ${\text{H}}_{3^{\text{′}}}$), 1.29 (app.dd, 1H, *J* = 8.4 Hz, ${\text{H}}_{3^{\text{′}}}$). ^13^**C NMR (CDCl**_3_**, 75 MHz**, δ**)**: 172.8 (C_1_), 147.1(C_6_), 135.5 (${\text{C}}_{1^{\text{′}}}$), 133.2 (C_7_), 131.7 (C_4_), 105.0 (C_5_), 64.7 (${\text{C}}_{5^{\text{′}}}$), 56.4 (C_8_), 49.0 (${\text{C}}_{3^{\text{′}}}$), 45.5 (${\text{C}}_{2^{\text{′}}}$), 40.7 (${\text{C}}_{4^{\text{′}}}$), 36.4 (C_2_), 31.3 (C_3_). **FT-IR (neat):** ν_max_ 3516 (ArOH), 1732 (C = O), 1608–1451 (C = C_Ar_). **UV (nm)**: λ_max_: 205. **HRMS (TOF MS, ES**+**):** *m/z* calcd for C_31_H_38_O_10_Na: 593.2363; found: 593.2371


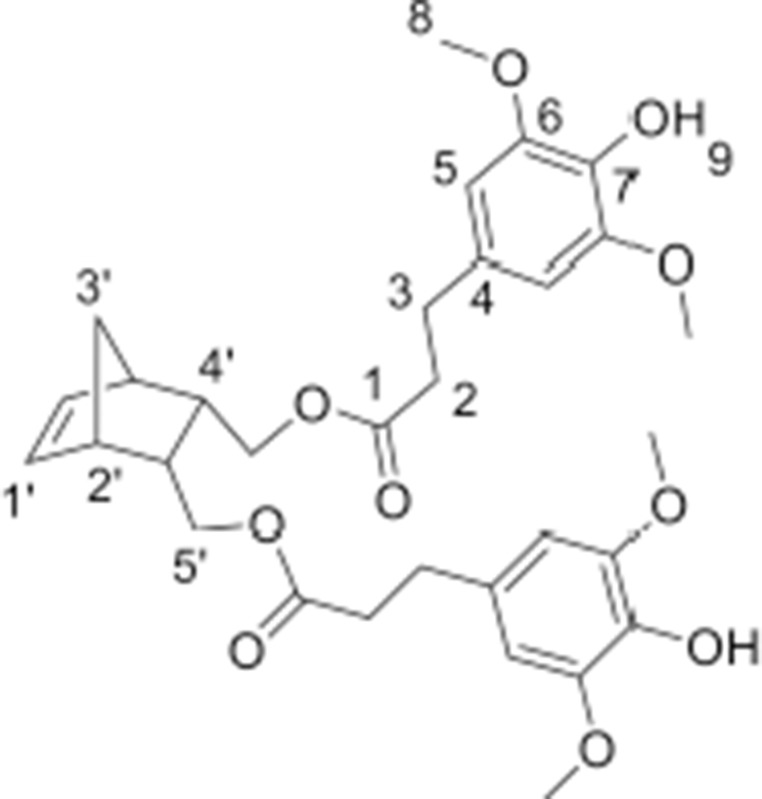


### General polymerization procedure [poly(NDF) and poly(NDS)]

In a typical experiment, a 25 mL flask is charged with monomer (**NDF**) or (**NDS**) (500 mg) under N_2_. CH_2_Cl_2_ (so that the final volume of the solution is 7.15 mL) and Grubbs 1^st^ generation or Hoveyda-Grubbs 2^nd^ generation catalyst solution in dichloromethane (8 mmol/L), were added with a syringe in sequence at different monomer to catalyst molar ratio ([M]/[C]) (from 25 to 1000). The mixture was stirred at room temperature for 3 h under N_2_. Ethyl vinyl ether (1 mL) was added to the mixture to quench the reaction and stirred for additional 10 min. The resulting polymer (poly(**NDF**) or poly(**NDS**)) was precipitated with an excess of diethyl ether at 0 °C. The polymer was filtered and washed with diethyl ether. A solid is obtained.

### General co-polymerization procedure [poly(N-*co*-NDF) and poly(N-*co*-NDS)]

In a typical experiment, a 25 mL flask is charged with monomers (**NDF**) or (**NDS**) and norbornene (**N**) (500 mg, molar ratio [**N**]/[**NDX**] from 0.33 to 9) under N_2_. CH_2_Cl_2_ (so that the final volume of the solution is 6 mL) and Grubbs 1st generation catalyst solution in dichloromethane (8 mmol/L, 2 mol%) were added with a syringe in sequence (total volume of solvent was 6 mL). Ethyl vinyl ether (1 mL) was added to the mixture to quench the reaction and stirred for additional 10 min. The resulting polymer [poly(**N**-*co*-**NDF**) or poly(**N**-*co*-**NDS**)] was precipitated with an excess of diethyl ether at 0 °C. The polymer was filtered and washed with diethyl ether. A solid is obtained.

### General hydrogenation procedure

The polymer is charged with *p*-toluenesulfonyl hydrazine (5 eq per double bond calculated) into a round-bottom flask. 10 mL of xylene are added with MOPS buffer (40 mg/mL). The reaction is heated at 135 °C. After 4 h, the reaction medium is allowed to cool down and then is filtered (Hahn, 1992). The polymer is precipitated into diethyl ether. Powders are obtained.

### DPPH analyses

Hundred and ninety microliter of homogeneous DPPH solution (200 μM) in ethanol are added to a well containing 10 μL of potential antiradical molecule solution in ethanol at different concentrations (from 300 μM to 9.3 μM). The reaction is followed by a microplate Multiskan FC, 1 scan every 5 min for 7.5 h at 515 nm. The use of different amounts of potential antioxidant give the EC_50_ value, which is describe as the efficient concentration needed to reduce half the initial population of DPPH. Each analysis was performed 4 times to have a mean value.

### Radical scavenging ability (RSA)

0.004 mmol of polymer are solubilized in 0.5 mL of chloroform. 0.1 mL of the polymer solution are dropped off in a quartz cell. Then the cell is placed under N_2_ for 1 h. We obtained a polymer film on the bottom of the cell. Two milliliters of a DPPH solution (0.1 mM) are added in the cell and the absorbance at 515 nm is measured at *t* = 0 and *t* = 3 h.

## Results and discussion

### Chemo-enzymatic synthesis of monomers

**Scheme 1:**
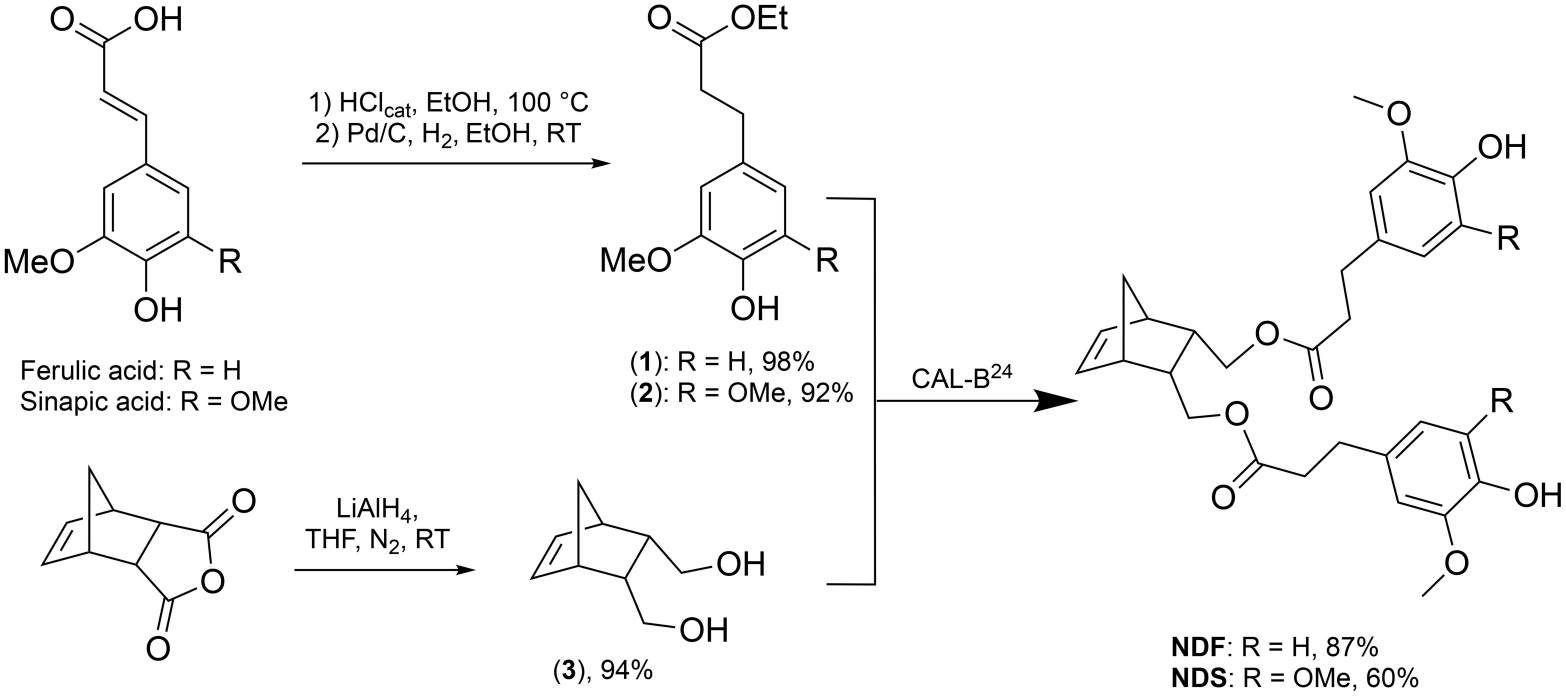
Chemo-enzymatic synthesis of **NDF** and **NDS** monomers.

The two norbornene-derived ester monomers bearing SHP groups, **NDF** and **NDS**, were prepared as described in Scheme 1. The first step consisted in synthesizing ethyl dihydroferulate (**1**, 98%) and ethyl dihydrosinapate (**2**, 92%) from ferulic acid and sinapic acid, respectively, through a two-step one-pot route involving Fisher esterification and palladium-mediated hydrogenation. 5-Norbornene-2endo,3endo-dimethanol (**3**) was then synthesized through the reduction of 5-norbornene-2,3-dicarboxylic anhydride in presence of LiAlH_4_ (94% yield). Finally, the solvent-free CAL-B-mediated transesterification (Pion et al., 2013) of **1** and **2** by **3** was performed to provide **NDS** (60%) and **NDF** (80%) monomers, respectively. One of the main advantages of using CAL-B in this synthesis is its inactivity toward phenols. Indeed, this particular selectivity renders unnecessary atom- and solvent-consuming protection/deprotection sequence. In addition, this bio-catalysis can be conducted under mild conditions (70 °C, solvent-free, under reduced pressure) and CAL-B can be readily recovered by filtration at the end of reaction. Finally, compared to the chemical synthesis of the similar monomer reported by Xue et al. (2007), this biocatalytic process is not only greener but also provides higher yields (60–80 vs. 37% (Xue et al., 2007)).

**Figure 2:**
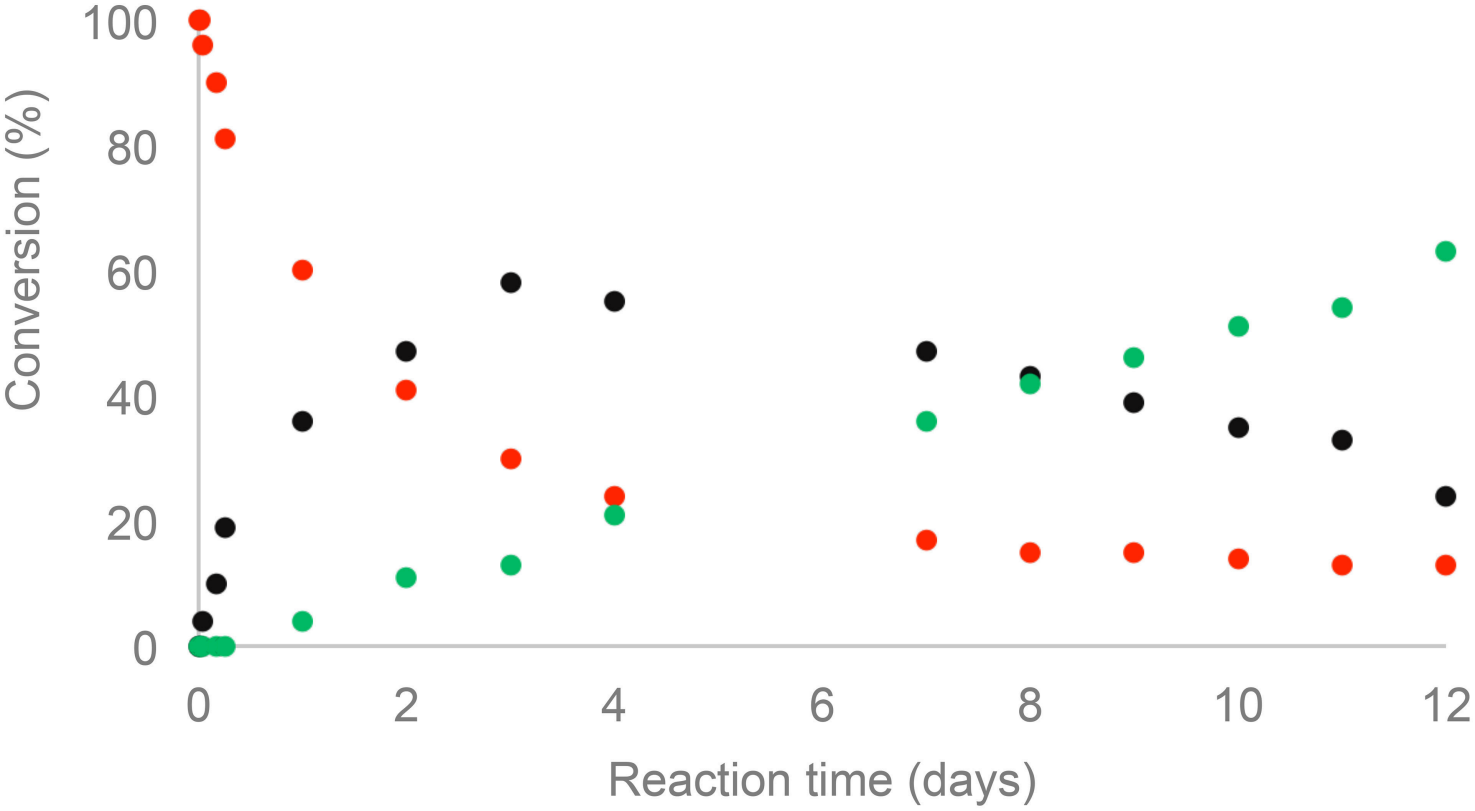
Kinetic monitoring by ^1^H NMR of transesterification of 5-norbornne-2endo,3endo-dimethanol (**3**) with ethyl dihydrosinapate (**2**): 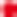
(**3**), 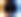
**NMS**, 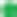
**NDS**.

The monitoring of ethyl dihydroferulate (**1**) transesterification with **3** using ^1^H NMR spectroscopy showed that it takes about 3 days to reach high yields, providing **NDF** in 87% yield after flash chromatography. In the case of **NDS**, for the same reaction time, a very low reactivity of ethyl dihydrosinapate (**2**) toward the enzyme was observed as **NDS** was obtained in 10% yield while up to 60% of norbornene mono-sinapate (**NMS**) was produced (Figure 2). It was shown that the reaction had to be run for more than 10 days to reach a **NDS** yield of 60%.

Both compounds were tested for their antiradical activity by using the DPPH method that determines the H-donor capacity of the antioxidant to quench the stable DPPH free radical, as previously reported (Reano et al., 2015). In this study, EC_50_ value corresponds to the amount of antioxidant (nmol) needed to reduce half of the initial population of DPPH radicals. The lower the EC_50_ value, the higher the antiradical activity. The total stoichiometry *n*_*tot*_ (number of DPPH molecules reduced by one molecule of antioxidant) of the monomers were also expressed in Table 1, according to: *n*_*tot*_ = *n*_DPPH_/2^*^EC_50_ [*n*_DPPH_: initial DPPH mole number (nmol)].

**Table 1:** Antiradical activity of **NDF** and **NDS**.

**Entry**	**Monomer**	**EC_50_ (nmol)**	***n_tot_***	***n_tot_/*phenol**
1	**NDF**	3.43 ± 0.05	5.5 ± 0.08	2.9 ± 0.04
2	**NDS**	2.29 ± 0.18	8.3 ± 0.65	4.4 ± 0.32
3	Irganox1010®	2.75 ± 0.28	6.9 ± 0.70	1.8 ± 0.18

As expected, data in Table 1 confirm the significant positive impact of methoxy group on both radical stability (+M, mesomeric effect) and antiradical activity since the higher the degree of methoxylation (**NDF** (1) vs. (**NDS** (2)), the lower the EC_50_ (**NDF** (EC_50_ = 3.43 nmol) vs. **NDS** (EC_50_ = 2.29 nmol)). **NDS** also exhibits a slightly higher antiradical activity than Iganox1010®. It is also noteworthy to mention that antiradical activity reported by the phenol numbers for **NDF** and **NDS** (i.e., 2 phenols for **NDF** and **NDS** and 4 phenols for Irganox1010®) are significantly higher than that of commercial Irganox1010®.

### ROMP (co-)polymerizations and characterization of the resulting (co-)polymers

#### Homo-polymerization of NDF and NDS

The ROMP homopolymerization of **NDF** and **NDS** antioxidant monomers ([M]) was performed using Grubs 1^st^ generation (GI), and Hoveyda-Grubbs 2^nd^ generation (HGII) catalysts ([C]) at various catalyst [M]/[C] molar ratios ranging from 25 to 1000. Because of the high reactivity of norbornene derivative in ROMP, room temperature was chosen to perform polymerization reactions while preventing insoluble high molecular weight polymers. Dichloromethane was used as solvent in invariable reaction medium volume of 7.15 mL under a low and steady nitrogen flow. All ROMP reactions were run for 3 h and followed by GPC in order to identify the best conditions (monomers nature, GI vs. HGII, [M]/[C]). The number molecular weight (*M*_*n*_) of polymers, degree of polymerization (*DP*_*n*_*)*, and polydispersity index (PDI) are summarized in Table 2.

**Table 2:** GPC and DSC data of synthesized polymers *via* ROMP.

**Entry**	**Monomer**	**Catalyst**	**[M]/[C]**	***M_*n*_* (kDa)**	**^a^*DP_*n*_***	**^a^PDI**	**^b^*T_*g*_*( °C)**
1	**NDF**	GI	25	7.2	14	2.0	32
2	**NDF**	GI	50	14.3	28	2.0	33
3	**NDF**	GI	200	22.0	43	2.5	36
4	**NDF**	GI	500	20.7	41	2.3	37
5	**NDF**	GI	1000	20.5	40	2.3	31
6	**NDF**	HGII	25	Insoluble	–	–	–
7	**NDF**	HGII	50	Insoluble	–	–	–
8	**NDF**	HGII	200	Insoluble	–	–	–
9	**NDF**	HGII	500	Insoluble	–	–	–
10	**NDF**	HGII	1000	Insoluble	–	–	–
11	**NDS**	GI	25	3.6	7	2.1	48
12	**NDS**	GI	50	4.5	8	1.8	54
13	**NDS**	GI	200	14.1	25	2.2	55
14	**NDS**	GI	500	15.0	26	2.0	54
15	**NDS**	GI	1000	20.5	40	2.1	56
16	**NDS**	HGII	25	Insoluble	–	–	–
17	**NDS**	HGII	50	Insoluble	–	–	–
18	**NDS**	HGII	200	Insoluble	–	–	–
19	**NDS**	HGII	500	Insoluble	–	–	–
20	**NDS**	HGII	1000	Insoluble	–	–	–

Polymerization conditions: 3 h at room temperature.

a GPC analyses: PLGel MixedD, 40 °C THF, 1 mL min^-1^, UV 280 nm, RI, calibration with polystyrene standards.

b *DSC data recording at 10 °C min^-1^ under nitrogen (60 mL min^-1^), value determined at the 2nd heating scan*.

Because of high molecular weights, ROMP polymerization performed with HGII catalyst became too viscous to be efficiently stirred after 15 min independently of [M]/[C] ratios (Table 2, entries 6–10 and 16–20). Resulting polymers could not be dissolved thus preventing any characterization by GPC.

On the basis of the results reported in Table 2, and as expected, a correlation between *M*_*n*_ and catalyst loading with GI polymerization was found (Table 2, entries 1–5 and 10–15). Indeed, *M*_*n*_ increases with the increase of [M]/[C] ratio under the same reaction conditions for both **NDF** (*M*_*n*_ entries 1 < 2 < 3) and **NDS** (*M*_*n*_ entries 11 < 12 < 13). ROMP being a chain growth type polymerization, the amount of reactive initiative species in chain initiation step increases with catalyst loading. Consequently, decreasing catalyst loading shifts the polymerization toward chain propagation and thus leads to higher molecular weight. In the case of **NDF**, an interesting plateau effect around 20.0 kDa values was observed from [M]/[C] = 200. Corresponding polymerization at higher ratios (i.e., 500 and 1000) resulted in *M*_*n*_ not exceeding 22.0 kDa. It is noteworthy to mention that a [M]/[C] ratio of 1,000 is needed to reach this *M*_*n*_ value with **NDS**. Finally, PDI values were in the range of 1.8–2.5, demonstrating a good homogeneity of the polymers chain length.

The thermal properties of the resulting polymers were investigated by thermogravimetric analysis (TGA) and differential scanning calorimetry (DSC) (see Table 2 and Electronic Supporting Information). TGA analyses of poly(**NDF**) and poly(**NDS**) revealed thermal stability (*T*_*d*50%_) higher than 350 °C in agreement with literature data on functionalized polynorbornene (Liu et al., 2007). No correlation was observed between thermal stability (*T*_*d*50%_) and degree of polymerization (*DP*_*n*_). DSC analyzes showed a 20 °C difference in *T*_*g*_ values between poly(**NDF**) and poly(**NDS**). Such difference is certainly due to the presence of an extra methoxy group on the aromatic ring in **NDS** that induces more rigidity to the monomer as previously reported (Janvier et al., 2017). Finally, it is noteworthy to mention that all polymers are amorphous and do not exhibit melting points (*T*_*m*_) on DSC.

#### Co-polymerization of norbornene (N) with NDF or NDS

**Scheme 2:**
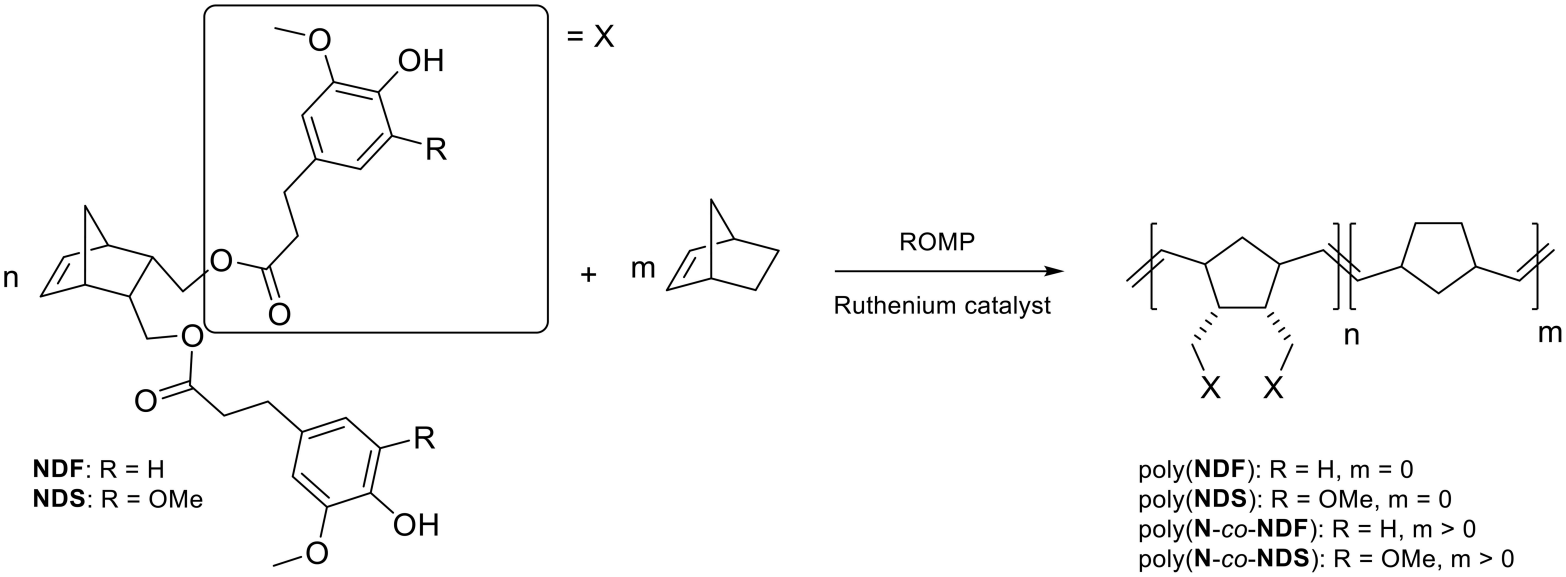
**NDF** or **NDS** (co-)polymerization via ROMP.

As the antioxidant activity is directly linked to the number of phenolic moieties, one could easily tune the antioxidant activity of the polymers by adjusting the number of SHP groups present per weight unit. Under these considerations, co-polymerization between antioxidant functionalized monomer (**NDF** or **NDS**) and non-active norbornene (**N**) has been performed (Scheme 2). Previous polymerization conditions were applied with molar ratio [**N**]/[**NDX**] varying from 1/3 to 9. Table 3 summarizes analytical data from GPC analyses.

**Table 3:** GPC characterization of crude copolymer via ROMP.

**Entry**	**Monomer**	**[N]/[NDX]_theo_**	**[N]/[NDX]^a^_exp_**	***^b^M_*n*_* (kDa)**	**^b^PDI**
1	**N-NDF**	1/3	Insoluble	13.8	2.1
2	**N-NDF**	1	Insoluble	9.6	2.2
3	**N-NDF**	3	Insoluble	7.7	3.1
4	**N-NDF**	9	n.d.	7.0	5.2
4^*^	N-NDF^*^	9	11.5^*^	15.8^*^	2.9^*^
5	**N-NDS**	1/3	Insoluble	14.9	3.1
6	**N-NDS**	1	Insoluble	11.8	3.8
7	**N-NDS**	3	Insoluble	8.4	5.7
8	**N-NDS**	9	n.d.	7.3	5.9
8^*^	N-NDS^*^	9	10.1^*^	25.2^*^	2.6^*^

Polymerization conditions: GI (molar ratio [M]/[C] = 50), 3h at room temperature.

a Calculated by 1H NMR.

b GPC analyses of crude polymers: PLGel MixedD, 40 °C THF, 1 mL min^−1^, UV 280 nm, RI, calibration with polystyrene standards.

* *Data after acetone wash*.

**Figure 3:**
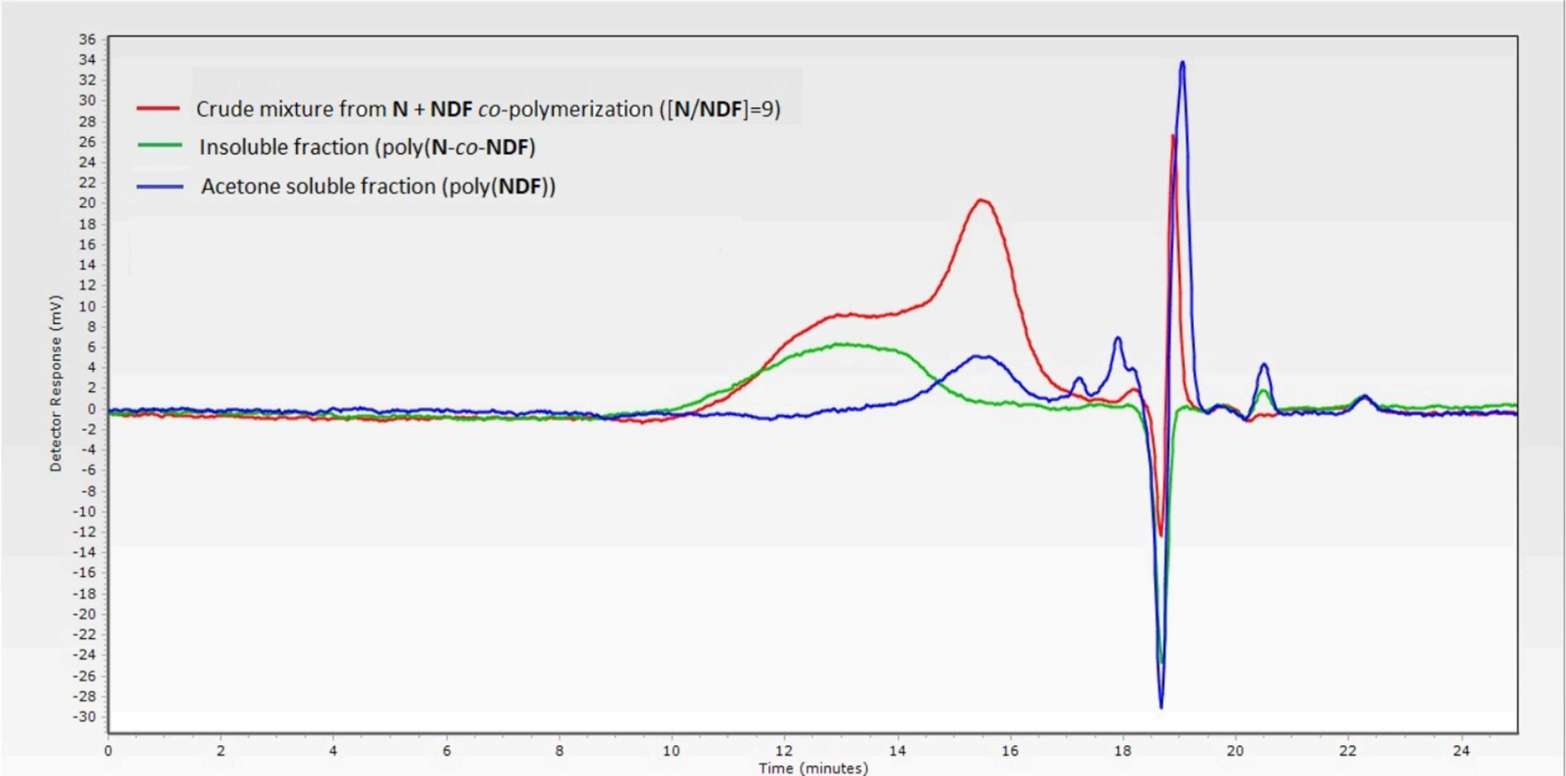
GPC traces of crude copolymerization (red), acetone-soluble polymers (blue) and acetone-insoluble polymers (green).

Increasing norbornene content during copolymerization strongly impacted the solubility of the resulting copolymers. Only a [**N**]/[**NDX**] ratio of 9 (Table 3, entries 4 and 8) provided soluble polymers after removing the catalyst by precipitation in diethyl ether. Therefore, GPC analyses were carried out directly on crude reaction medium before precipitation to measure *M*_*n*_ and PDI values. These results showed distinctly that the decrease of **NDF**/**NDS** monomer content results in decreasing *M*_*n*_ values down to 7.0 kDa, while PDI value skyrockets over 5.0 (Table 3, entry 1 vs. entry 4). GPC trace investigation reveals a bimodal distribution explaining the significant increase of PDI values while inducing irremediably inaccurate *M*_*n*_ estimations (Figure 3 — red trace). Judging from this observation, it was concluded that co-polymerization probably gave two different species of polymers.

**Scheme 3:**
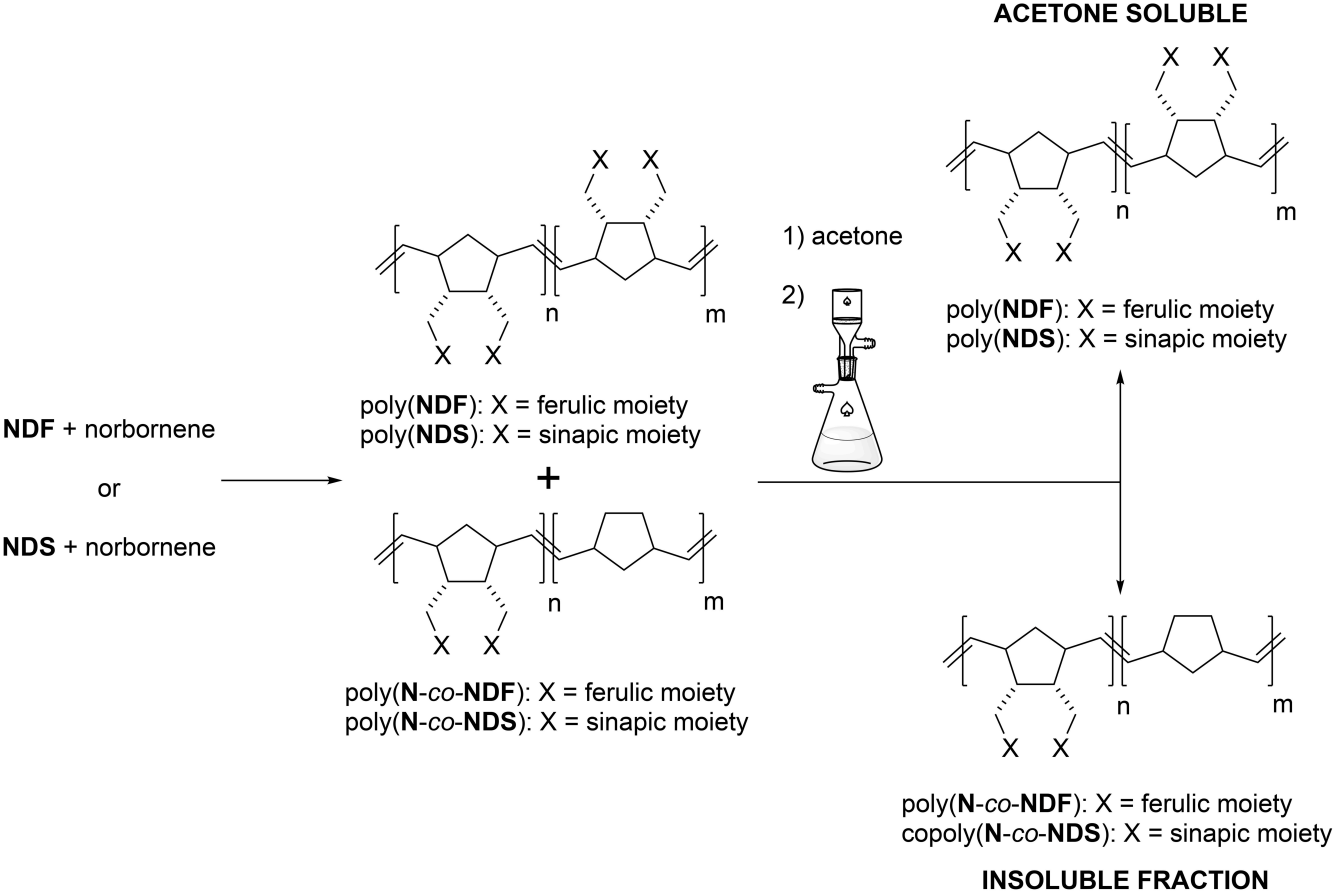
Acetone wash method to separate poly(**NDX**) from poly(**N**-*co*-**NDX**).

Advantageously, we found out that a simple acetone wash allowed the separation of the two types of polymers (Scheme 3), which were then analyzed separately by GPC, revealing more acceptable *M*_*n*_ and PDI values (Figure 3—green trace, Table 3 — entries 4^*^ and 8^*^, Table 4). By combining GPC traces (Figure 3—blue trace) and ^1^H NMR spectroscopy data (see ESI), it was established that the soluble polymers present in the acetone fraction correspond to homo-polymers poly(**NDF**) or poly(**NDS**), while the insoluble polymer was a copolymer of norbornene (**N**) and **NDF** or **NDS**, poly(**N**-*co*-**NDF**) and poly(**N**-*co*-**NDS**), respectively.

**Table 4:** GPC characterization of (co-)polymers after acetone wash.

**Entry**	**Monomers**	**[N]/[NDX]_theo_**	**Polymers**	**[N]/[NDX]^a^_exp_**	***^b^M_*n*_* (kDa)**	**^b^PDI**
1	**N** + **NDF**	9	Poly(**N**-*co*-**NDF**)	11.5	15.8	2.9
			Poly(**NDF**)	–	6.5	1.8
2	**N** + **NDS**	9	Poly(**N**-*co*-**NDS**)	10.1	25.2	2.6
			Poly(**NDS**)	–	2.6	1.4

Polymerization conditions: GI (molar ratio [M]/[C] = 50), DCM (6 mL), 3h at room temperature.

a Calculated by 1H NMR.

b *GPC analyses of crude polymers: PLGel MixedD, 40 °C THF, 1 mL min^-1^, UV 280 nm, RI, calibration with polystyrene standards*.

#### Hydrogenation

**Scheme 4:**
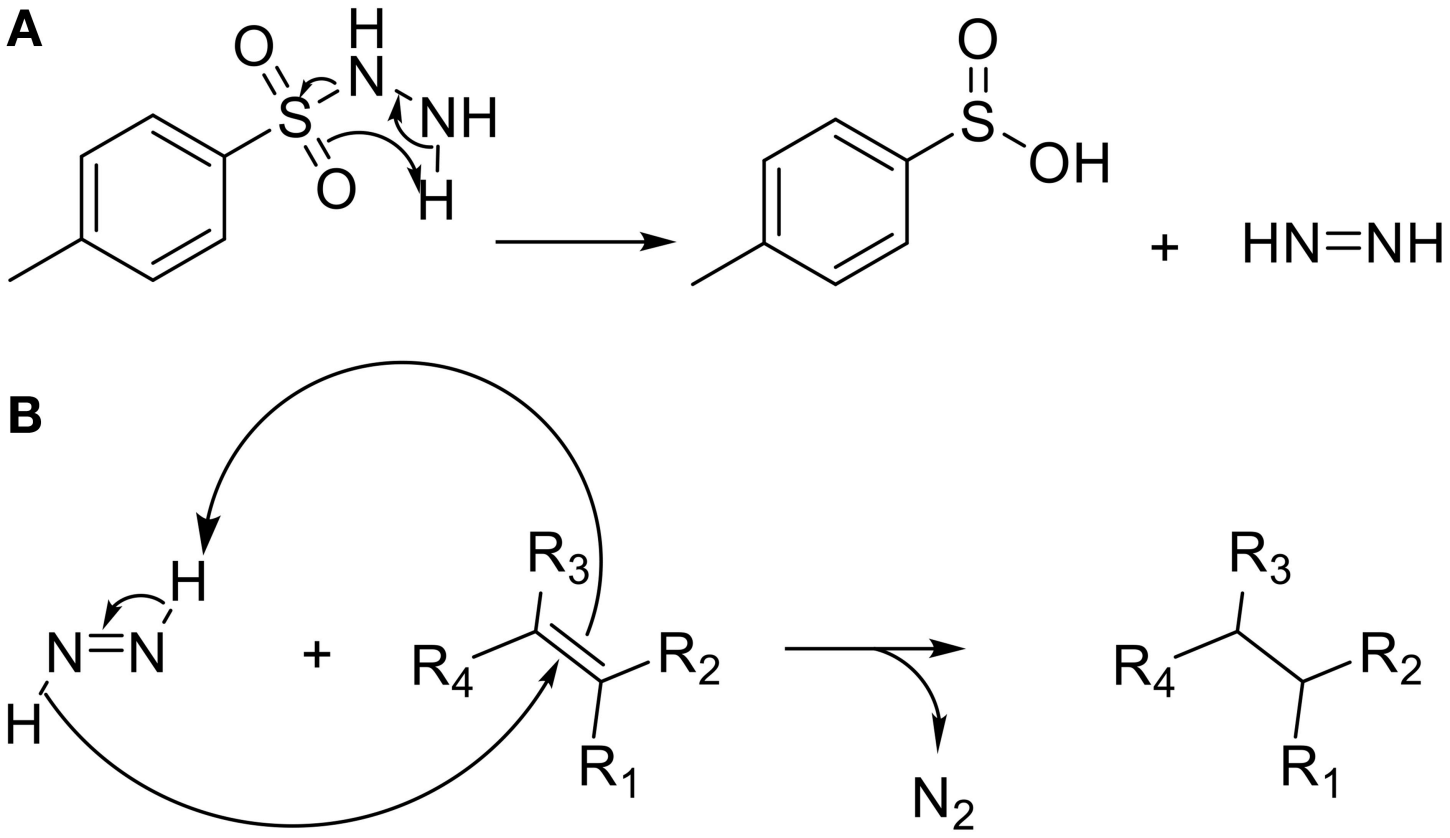
Mechanism of hydrogenation involving *p*-toluenesulfonylhydrazine (*p*-TSH).

The unsaturated polymers obtained by ROMP being susceptible to oxidation and less thermally stable than their saturated counterparts (Linwood et al., 1989; Yoon et al., 2012) poly(**NDF**), poly(**NDS**), poly(**N**-*co*-**NDF**) and poly(**N**-*co*-**NDS**) have been submitted to hydrogenation in presence of *p*-toluenesulfonylhydrazine (*p*-TSH) as described by Xue et al. (2008) (Scheme 4). An excess of *p*-TSH (5 eq. per double bound) was used as a precursor to generate diimide and *p*-TSH *in situ* (a). The generated diimide reduced selectively apolar double bound, leaving the aromatic rings of the SHP untouched, and gave off N_2_ gas (b).

**Figure 4:**
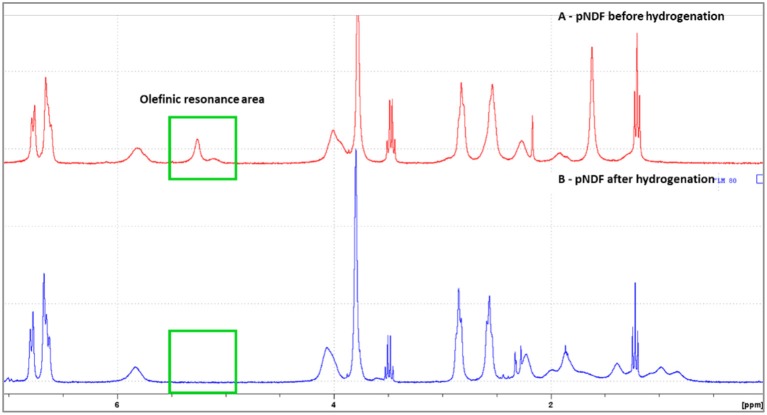
^1^H NMR spectra (300 MHz) of poly(**NDF**) before (A-red) and after hydrogenation (B-blue).

It is noteworthy to mention that the co-generated *p*-TSH can lead to chemical degradation of polymers, thus resulting in a decrease of *M*_*n*_ (Yoon et al., 2011). Therefore, basic reagents such as MOPs were used to neutralize *p*-toluenesulfinic acid (Table 5). Under these conditions, all unsaturations were successfully removed as evidenced by the absence of the olefinic peaks at 5.0–5.7 ppm in the ^1^H NRM spectrum of the resulting polymers (Figure 4). Unfortunately, all our attempts to develop a greener hydrogenation procedure remained unsuccessful.

**Table 5:** GPC characterization of hydrogenate polymer.

**Entry**	**Polymer**	**[N]/[NDX]_theo_**	***^a^M_*n*_* (kDa)**
1	poly-H-(**NDF**)	–	18.5
2	poly-H-(**NDS**)	–	13.5
3	poly-H-(**N**-*co*-**NDF**)	9	12.0
4	poly-H-(**N**-*co*-**NDS**)	9	19.0

a *GPC analyses of crude polymers: PLGel MixedD, 40 °C THF, 1 mL min^-1^, UV 280 nm, RI, calibration with polystyrene standards*.

### Antiradical activity determination using DPPH analysis

In DPPH analysis, radical scavenging ability (%RSA) is linked to the ability of SHP molecules to scavenge α,α-diphenyl-β-picrylhydrazyl (DPPH) free radical (Blois, 1958; Brand-Williams et al., 1995). In this study, %RSA was defined as the rate of absorption loss for DPPH solution measured at 515 nm after shaking for 3 h in presence of equimolar amount of poly-H-(**NDF**), poly-H-(**NDS**), poly-H-(**N**-*co*-**NDF**) or poly-H-(**N**-*co*-**NDS**) copolymers films. Only copolymers with [**N**]/[**NDX**] ratio of 9 were submitted to the RSA test as the others copolymers were poorly soluble.

Table 6 shown RSA < 2% for polynorbornene. Bearing no SHP group, this very low activity was due only to auto-reduction of DPPH in solution during analysis time (3 h). Homopolymers poly-H-(**NDF**) and poly-H-(**NDS**) exhibited interesting activities as both reduced more than 80% of the initial population of free radical. In addition, as already observed at the monomer level, poly-H-(**NDS**) is more antioxidant than poly-H-(**NDF**). Logically, due to a lower SHP content, the RSA of copolymers poly-H-(**N**-*co*-**NDF**) and poly-H-(**N**-*co*-**NDS**) is lower than that of poly-H-(**NDF**) and poly-H-(**NDS**), respectively. However, it is noteworthy to mention that a 9-fold decrease of SHP content (poly-H-(**N**-*co*-**NDF**) vs. poly-H-(**NDF**), poly-H-(**N**-*co*-**NDS**) vs. poly-H-(**NDS**)) only resulted in a *ca*. 2-fold decrease in RSA, demonstrating that not only the radical scavenging activity is not proportional to the SHP content, but also that only a relatively small SHP content is needed to provide a potent antioxidant activity to the polymers.

**Table 6:** Radical scavenging ability of polymers and copolymers.

**Entry**	**Polymer**	**[N]/[NDX]_theo_**	***M_*n*_* (kDa)**	**% RSA**	**% RSA/phenol**
1	poly-H-(**N**)	–	–	2.0 ± 0.5	–
2	poly-H-(**NDF**)	–	18.5	82.3 ± 1.9	1.1 ± 0.03
3	poly-H-(**NDS**)	–	13.5	91.8 ± 1.3	1.9 ± 0.03
4	poly-H-(**N**-*co*-**NDF**)	9	12.0	40.0 ± 1.5	2.7 ± 0.10
5	poly-H-(**N**-*co*-**NDS**)	9	19.0	33.8 ± 0.9	1.4 ± 0.04

## Conclusions

Biobased antioxidant monomers bearing SHP were successfully synthesized from renewable ferulic and sinapic acids using a sustainable process involving a lipase-mediated transesterification. These monomers were then (co-)polymerized using ROMP in presence of Grubbs 1^st^ generation catalyst. Thanks to a very simple procedure involving an acetone wash, the homo- and copolymers where easily separated and fully characterized by GPC, ^1^H NMR, DSC and TGA before being submitted to hydrogenation. The antioxidant activity of the resulting saturated polymers was assessed by DPPH analysis and revealed a high antioxidant activity for both homo- and copolymers, and this even for copolymers with SHP-bearing monomer contents as low as 8%. This work demonstrates that the combination of biocatalysis and biobased sterically hindered phenolics such as ferulic and sinapic acids offers a greener and more efficient alternative to those already reported in the literature that uses fossil-based SHP and classic chemical synthesis.

## Author contributions

FA: conceived and managed the research. FD-N and LM: performed the chemo-enzymatic reactions and the characterizations; LH, FR: provided the technical guidelines. FA, LH, and FR: reviewed the results and provided the technical guidelines. FA and LH: wrote and drafted the article. FA and SD: reviewed and approved the article.

### Conflict of interest statement

The authors declare that the research was conducted in the absence of any commercial or financial relationships that could be construed as a potential conflict of interest.
