# Supplementary material for: Biocatalytic Synthesis and Polymerization via ROMP of New Biobased Phenolic Monomers: A Greener Process toward Sustainable Antioxidant Polymers
Source: Front Chem. 2017 Dec 22;5:126. doi: 10.3389/fchem.2017.00126 (PMC5744638; doi:10.3389/fchem.2017.00126)

Biocatalytic synthesis and polymerization via ROMP of new biobased phenolic monomers: a greener process towards sustainable antioxidant polymers

Diot-Néant,[§a,b] F.; Migeot,[§a] L.; Hollande,[a,c] L.; Reano,[a,c] A.F.; Domenek,[c] S.; Allais,*[a,d] F.

[a] Chaire ABI, AgroParisTech, CEBB 3 rue des Rouges Terres 51110 Pomacle, France
[b] The George and Josephine Butler Laboratory for Polymer Research, Department of Chemistry, University of Florida Gainesville, Florida, 32611-7200, USA
[c] UMR GENIAL, AgroParisTech, INRA, Université Paris-Saclay, Avenue des Olympiades, 91300 Massy, France
[d] UMR 782 GMPA, AgroParisTech, INRA, Université Paris-Saclay, Avenue Lucien Brétignières 78850 Thiverval-Grignon, France
[§] these authors contributed equally to this work

florent.allais@agroparistech.fr

TABLE OF CONTENT

1. ^{1}H and ^{13}C NMR spectra of Norbornene diferulate ($CDCl_3$) S2
2. ^{1}H and ^{13}C NMR spectra of norbornene disinapate ($CDCl_3$) S4
3. FT-IR spectra of Norbornene diferulate (**NDF**) S6
4. FT-IR spectra of Norbornene disinapate (**NDS**) S7
5. HRMS spectra of Norbornene diferulate (**NDF**) S8
6. HRMS spectra of Norbornene disinapate (**NDS**) S9
7. DPPH analysis (EC_{50}) of Norbornene diferulate (**NDF**) S10
8. DPPH analysis (EC_{50}) of Norbornene disinapate (**NDS**) S11
9. ^{1}H NMR spectra of poly(**NDF**) S12
10. ^{1}H NMR spectra of poly(**NDS**) S13
11. TGA analysis of poly(**NDF**) S14
12. TGA analysis of poly(**NDS**) S15
13. DSC analysis of poly(**NDF**) S16
14. DSC analysis of poly(**NDS**) S17
15. GPC analysis of poly(**N**-*co*-**NDF**) S18
16. GPC analysis of acetone wash method to separate poly(**NDF**) and poly(**N**-*co*-**NDF**) S19
17. ^{1}H NRM spectra of poly(**N**-*co*-**NDF**) S20
18. GPC analysis of poly(**N**-*co*-**NDS**) S21
19. GPC analysis of acetone wash method to separate poly(**NDS**) and poly(**N**-*co*-**NDS**) S22
20. ^{1}H NMR spectra of poly(**N**-*co*-**NDS**) S23
21. TGA analysis of poly(**N**-*co*-**NDF**) S24
22. TGA analysis of poly(**N**-*co*-**NDS**) S25

1. ^{1}H and ^{13}C NMR spectra of Norbornene diferulate ($CDCl_3$)

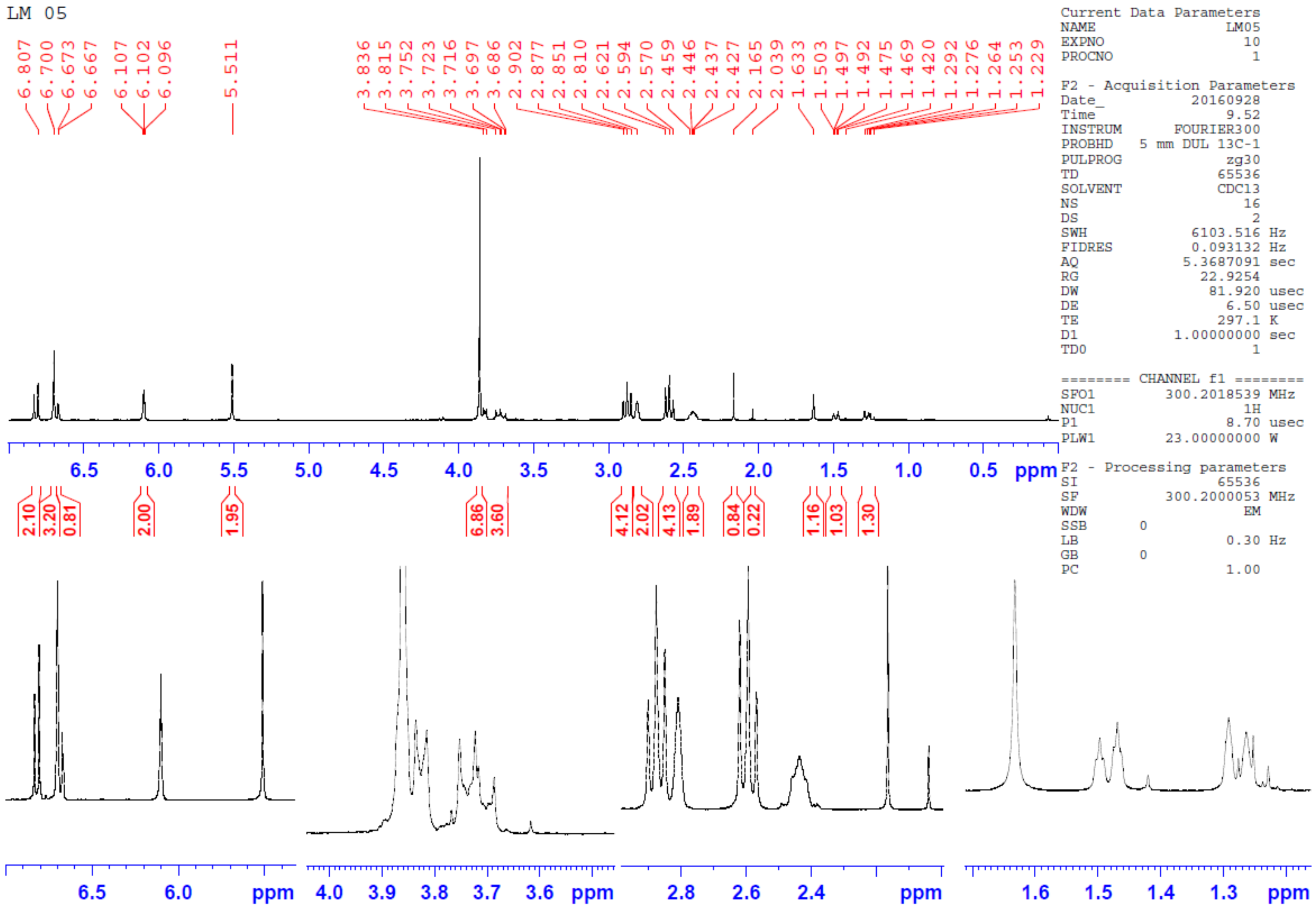

Norbornene diferulate

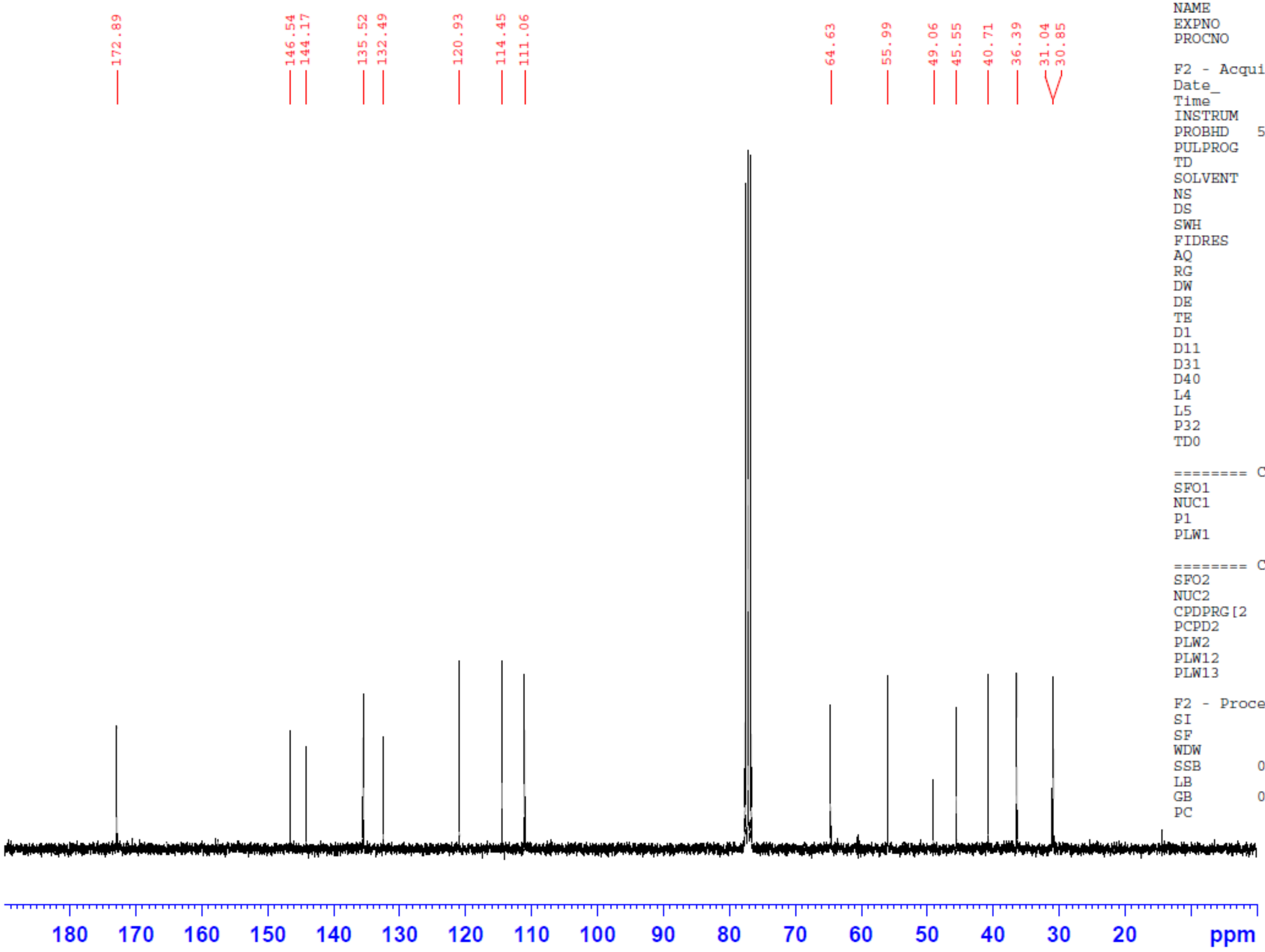


```
Current Data Parameters
NAME             LM05 F2
EXPNO                 11
PROCNO                 1

F2 - Acquisition Parameters
Date_           20160926
Time               17.51
INSTRUM       FOURIER300
PROBHD   5 mm DUL 13C-1
PULPROG          zgpg30
TD                 65536
SOLVENT            CDCl3
NS                  1024
DS                     4
SWH            24414.063 Hz
FIDRES          0.372529 Hz
AQ             1.3421773 sec
RG               501.187
DW                20.480 usec
DE                  6.50 usec
TE                 298.2 K
D1            2.00000000 sec
D11           0.03000000 sec
D31           0.00001300 sec
D40           0.00439029 sec
L4                    37
L5                    53
P32                98.00 usec
TD0                    1

======== CHANNEL f1 ========
SFO1          75.4928982 MHz
NUC1                 13C
P1                 13.00 usec
PLW1         22.00000000 W

======== CHANNEL f2 ========
SFO2         300.2012008 MHz
NUC2                  1H
CPDPRG[2         waltz16
PCPD2              98.00 usec
PLW2         23.00000000 W
PLW12         0.30000001 W
PLW13         0.23823000 W

F2 - Processing parameters
SI                 32768
SF            75.4853411 MHz
WDW                   EM
SSB      0
LB                  1.00 Hz
GB       0
PC                  1.40
```

2. ^{1}H and ^{13}C NMR spectra of norbornene disinapate ($CDCl_3$)

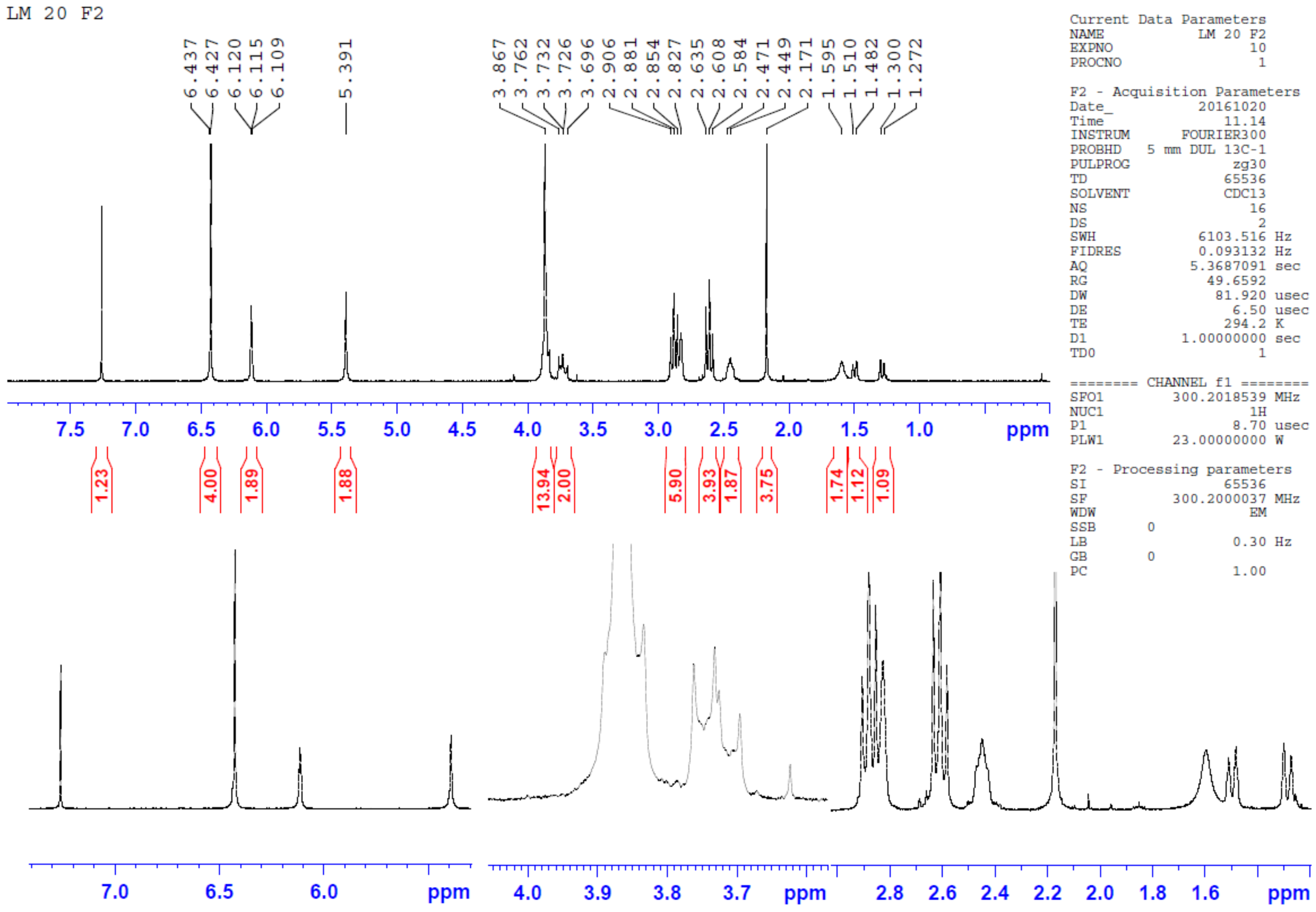

Norbornene DiSynapate

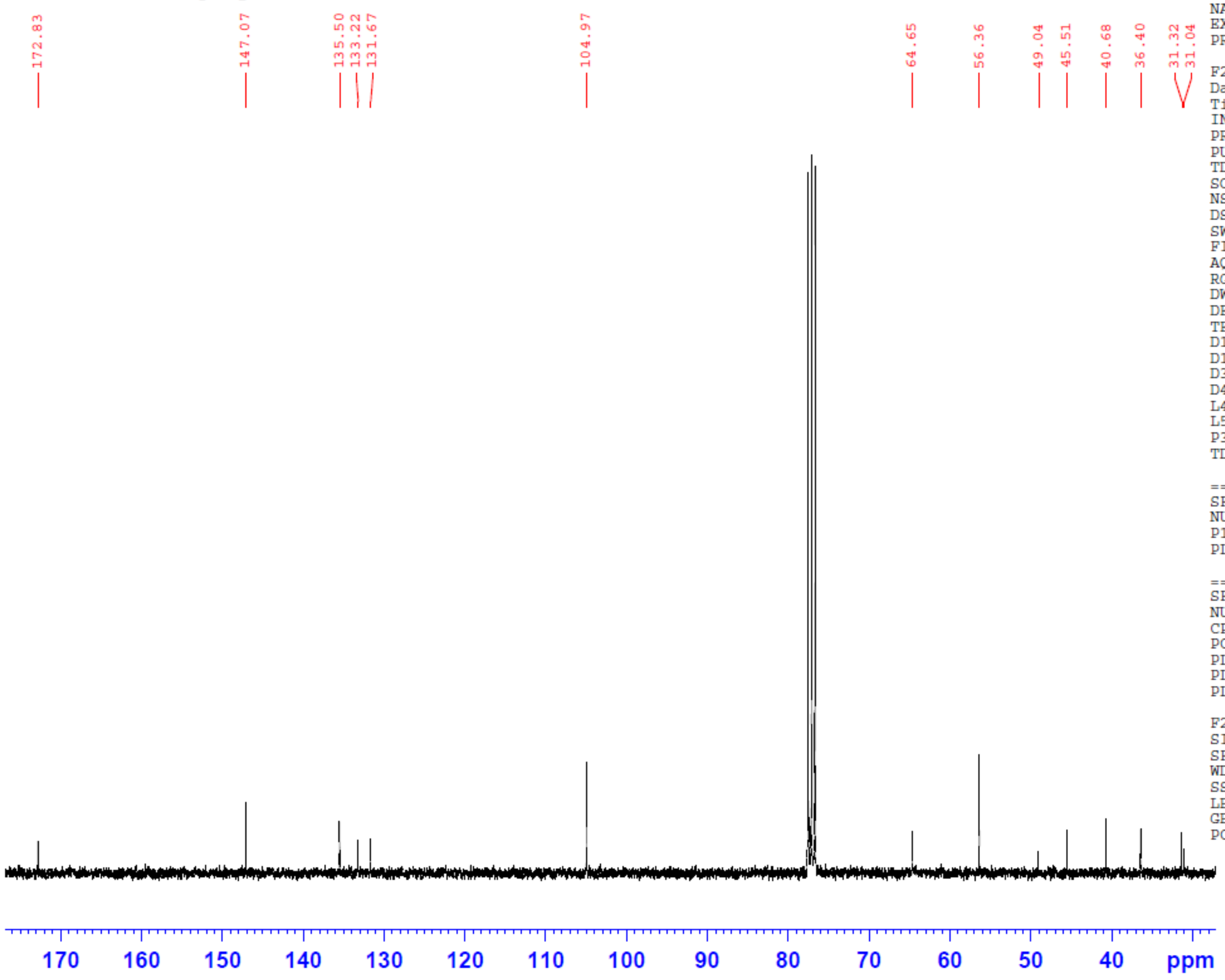


```
Current Data Parameters
NAME              LM 20 F2
EXPNO                   11
PROCNO                   1

F2 - Acquisition Parameters
Date_             20161020
Time                 19.07
INSTRUM         FOURIER300
PROBHD     5 mm DUL 13C-1
PULPROG            zgpg30
TD                  65536
SOLVENT             CDCl3
NS                   1024
DS                      4
SWH             24414.063 Hz
FIDRES           0.372529 Hz
AQ              1.3421773 sec
RG                501.187
DW                 20.480 usec
DE                   6.50 usec
TE                  295.4 K
D1             2.00000000 sec
D11            0.03000000 sec
D31            0.00001300 sec
D40            0.00439029 sec
L4                     37
L5                     53
P32                 98.00 usec
TD0                     1

======== CHANNEL f1 ========
SFO1           75.4928982 MHz
NUC1                  13C
P1                  13.00 usec
PLW1          22.00000000 W

======== CHANNEL f2 ========
SFO2          300.2012008 MHz
NUC2                   1H
CPDPRG[2          waltz16
PCPD2               98.00 usec
PLW2          23.00000000 W
PLW12          0.30000001 W
PLW13          0.23823000 W

F2 - Processing parameters
SI                  32768
SF             75.4853430 MHz
WDW                    EM
SSB      0
LB                   1.00 Hz
GB       0
PC                   1.40
```

3. FT-IR spectra of Norbornene diferulate (**NDF**)

Agilent Resolutions Pro

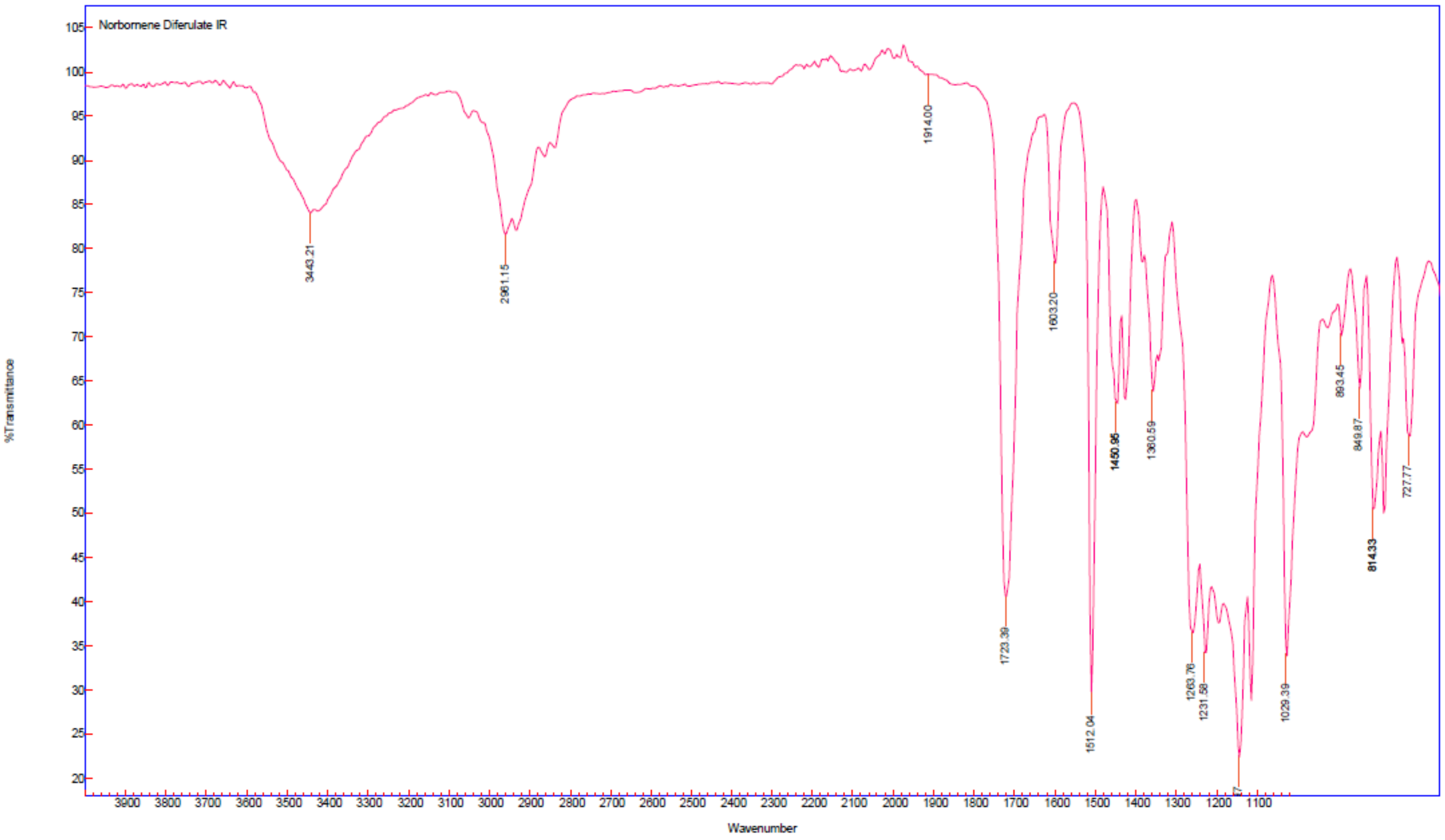

Name
Norbornene Diferulate IR

4. FT-IR spectra of Norbornene disinapate (**NDS**)

Agilent Technologies

Sample ID:	LM NDS	Method Name:	ALF generale
Sample Scans:	32	User:	Admin
Background Scans:	32	Date/Time:	3/20/2017 11:01:19AM
Resolution:	8 cm-1	Range:	4,000.00 - 650.00
System Status:	Good	Apodization:	Happ-Genzel
File Location:	C:\Program Files\Agilent\MicroLab PC\Results\LM NDS_2017-03-20T11-02-38.a2r		

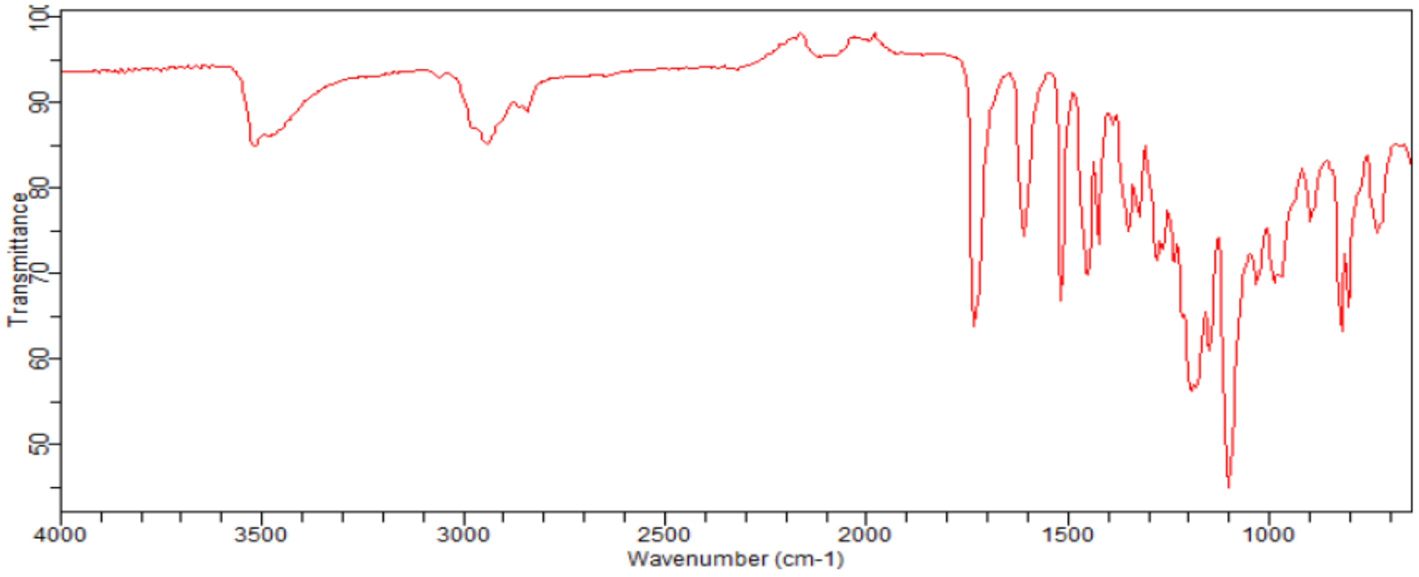

5. HRMS spectra of Norbornene diferulate (**NDF**)

Elemental Composition Report

Page 1

Single Mass Analysis

Tolerance = 5.0 PPM / DBE: min = -1.5, max = 50.0
Element prediction: Off
Number of isotope peaks used for i-FIT = 3

Monoisotopic Mass, Even Electron Ions
285 formula(e) evaluated with 1 results within limits (up to 50 closest results for each mass)
Elements Used:
C: 29-29 H: 0-200 O: 6-14 Na: 0-3 Al: 0-1 39K: 0-1 90Zr: 0-1

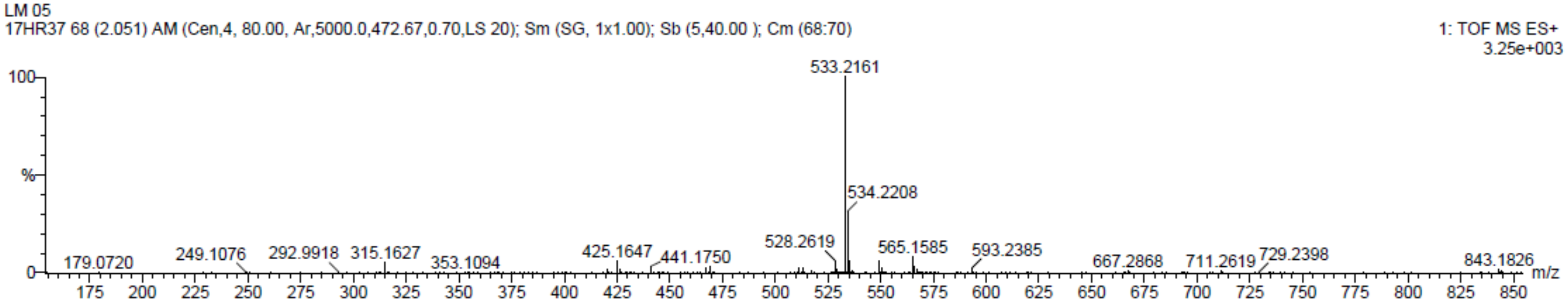

Minimum:				-1.5		
Maximum:		5.0	5.0	50.0		
Mass	Calc. Mass	mDa	PPM	DBE	i-FIT	Formula
533.2161	533.2151	1.0	1.9	12.5	2.9	C29 H34 O8 Na

6. HRMS spectra of Norbornene disinapate (**NDS**)

Elemental Composition Report

Single Mass Analysis

Tolerance = 5.0 PPM / DBE: min = -1.5, max = 50.0
Element prediction: Off
Number of isotope peaks used for i-FIT = 3

Monoisotopic Mass, Even Electron Ions
281 formula(e) evaluated with 1 results within limits (up to 50 closest results for each mass)
Elements Used:
C: 31-31 H: 0-200 O: 6-14 Na: 0-3 Al: 0-1 39K: 0-1 90Zr: 0-1

LM 20
17HR36 131 (4.252) AM (Cen,4, 80.00, Ar,5000.0,472.67,0.70,LS 20); Sm (SG, 1x1.00); Sb (5,40.00); Cm (131:134)
1: TOF MS ES+
9.46e+002

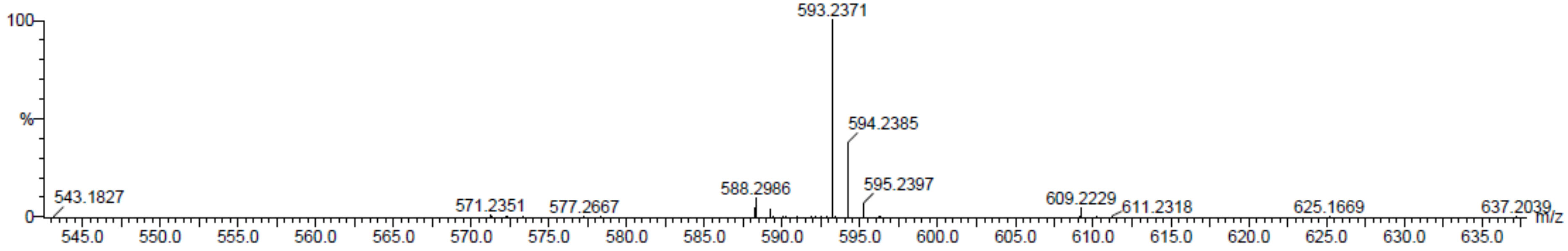

Minimum:				-1.5		
Maximum:		5.0	5.0	50.0		
Mass	Calc. Mass	mDa	PPM	DBE	i-FIT	Formula
593.2371	593.2363	0.8	1.3	12.5	1.6	C31 H38 O10 Na

7. DPPH analysis (EC_{50}) of Norbornene diferulate (**NDF**)

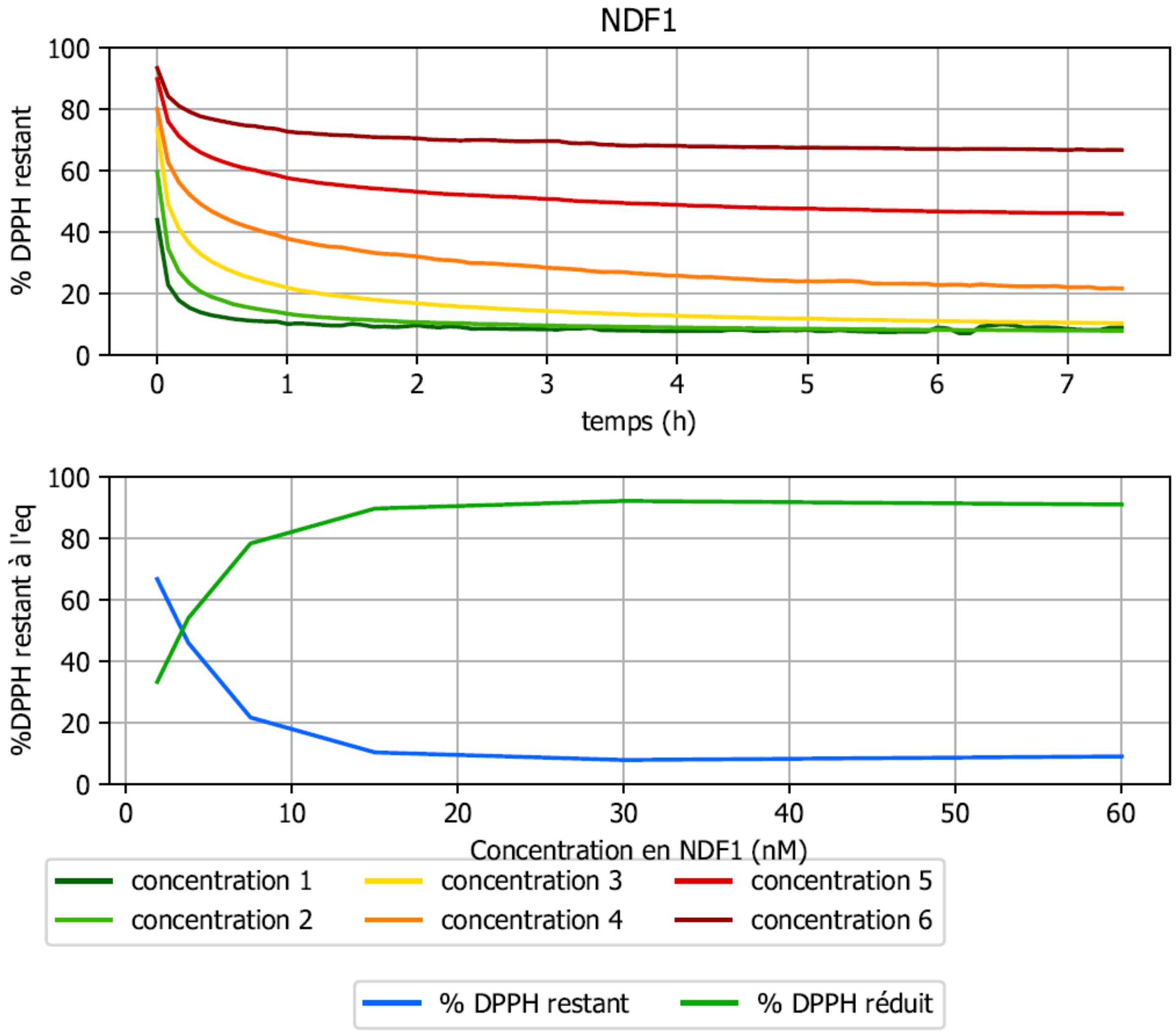

8. DPPH analysis (EC_{50}) of Norbornene disinapate (**NDS**)

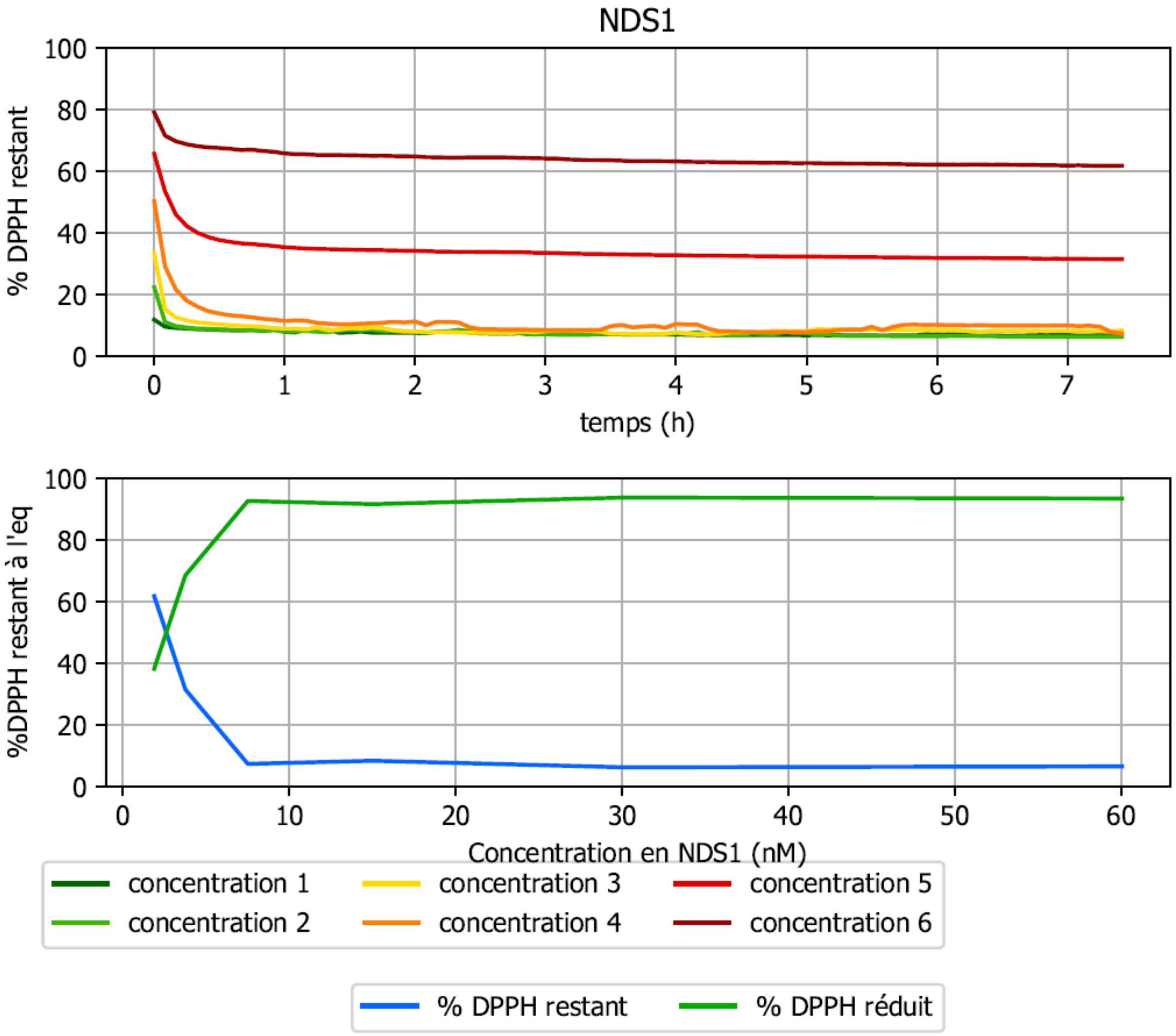

9. ^{1}H NMR spectra of poly(**NDF**)

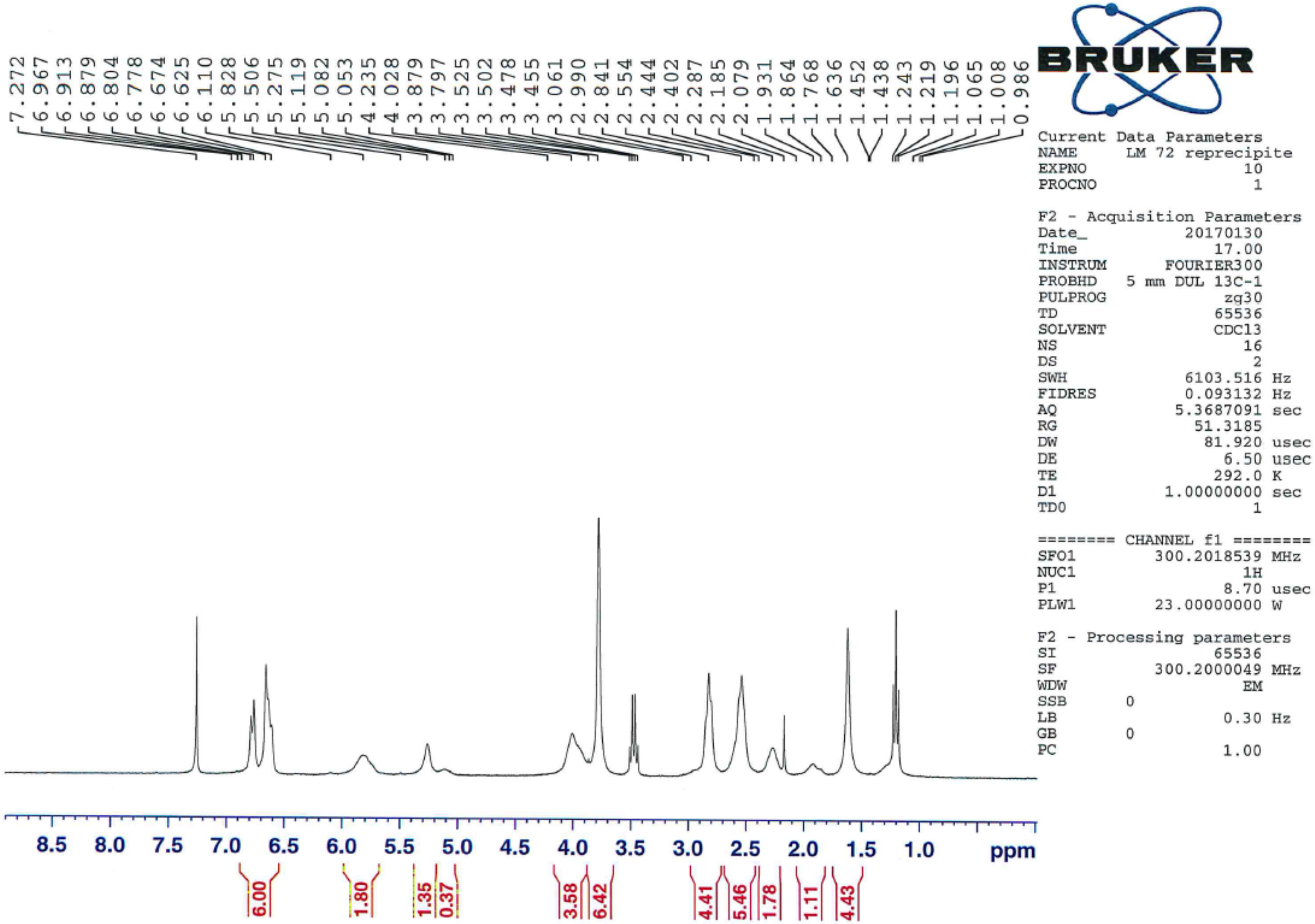

10. ^{1}H NMR spectra of poly(**NDS**)

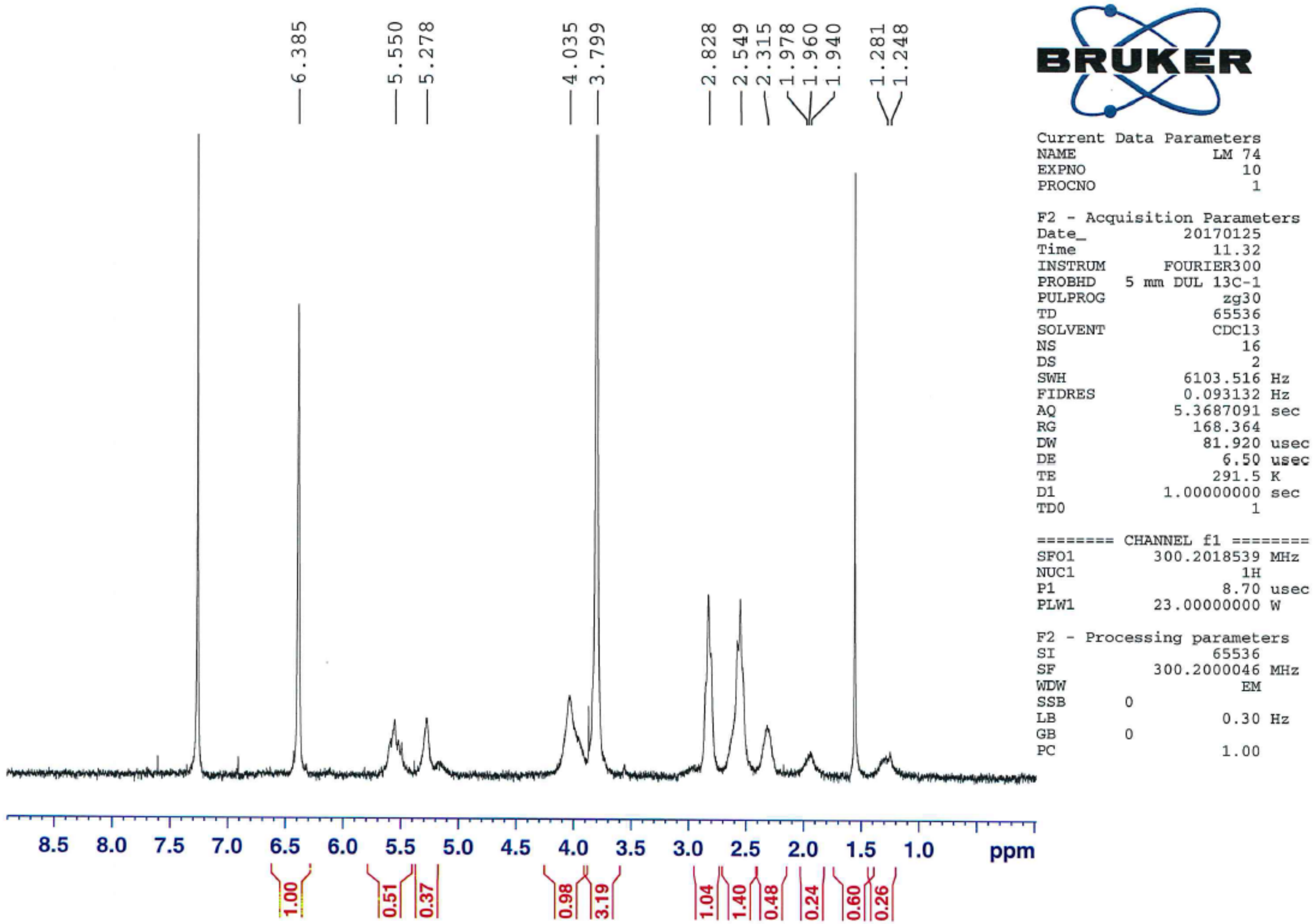

11. TGA analysis of poly(**NDF**)

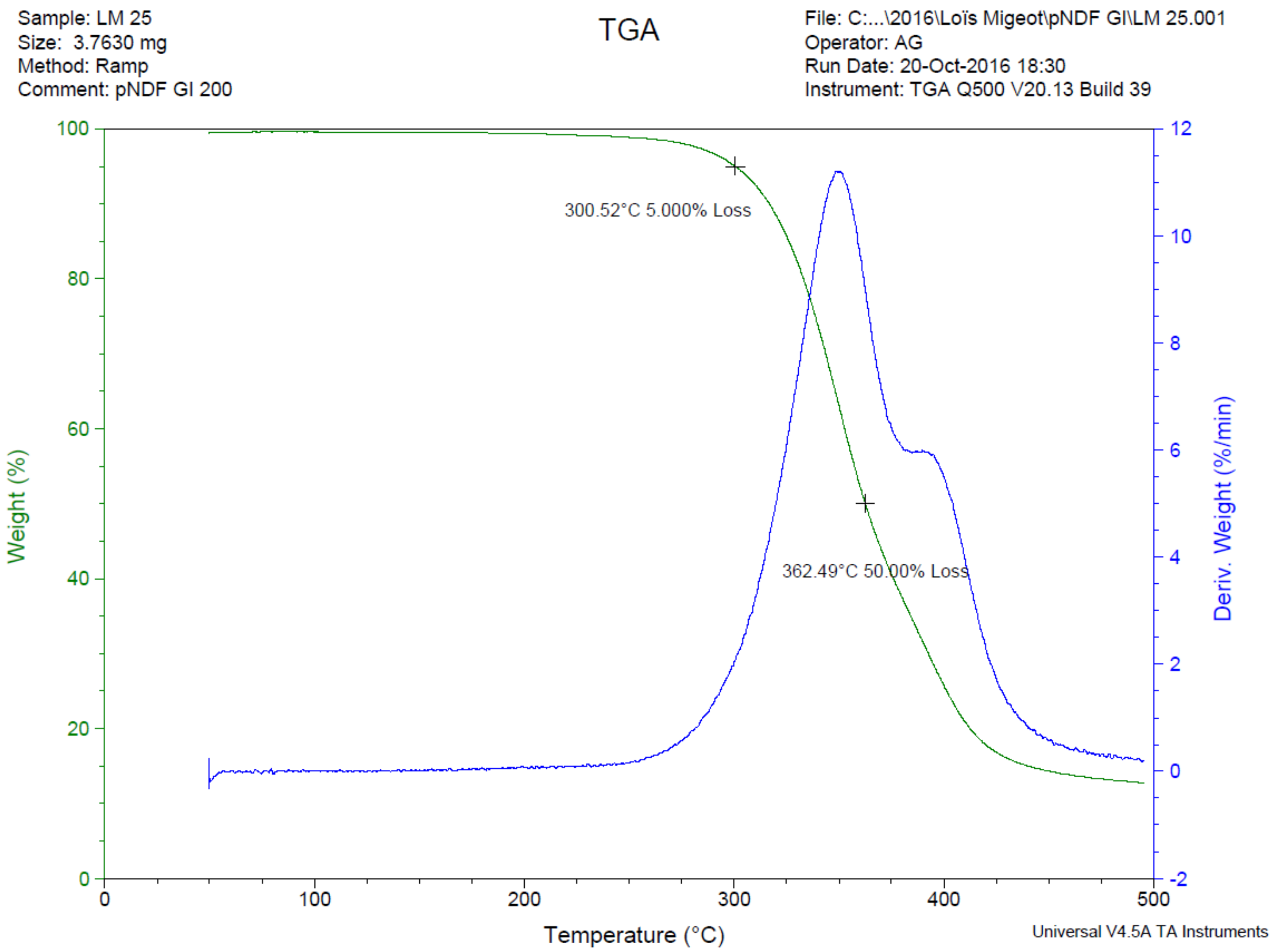

12. TGA analysis of poly(**NDS**)

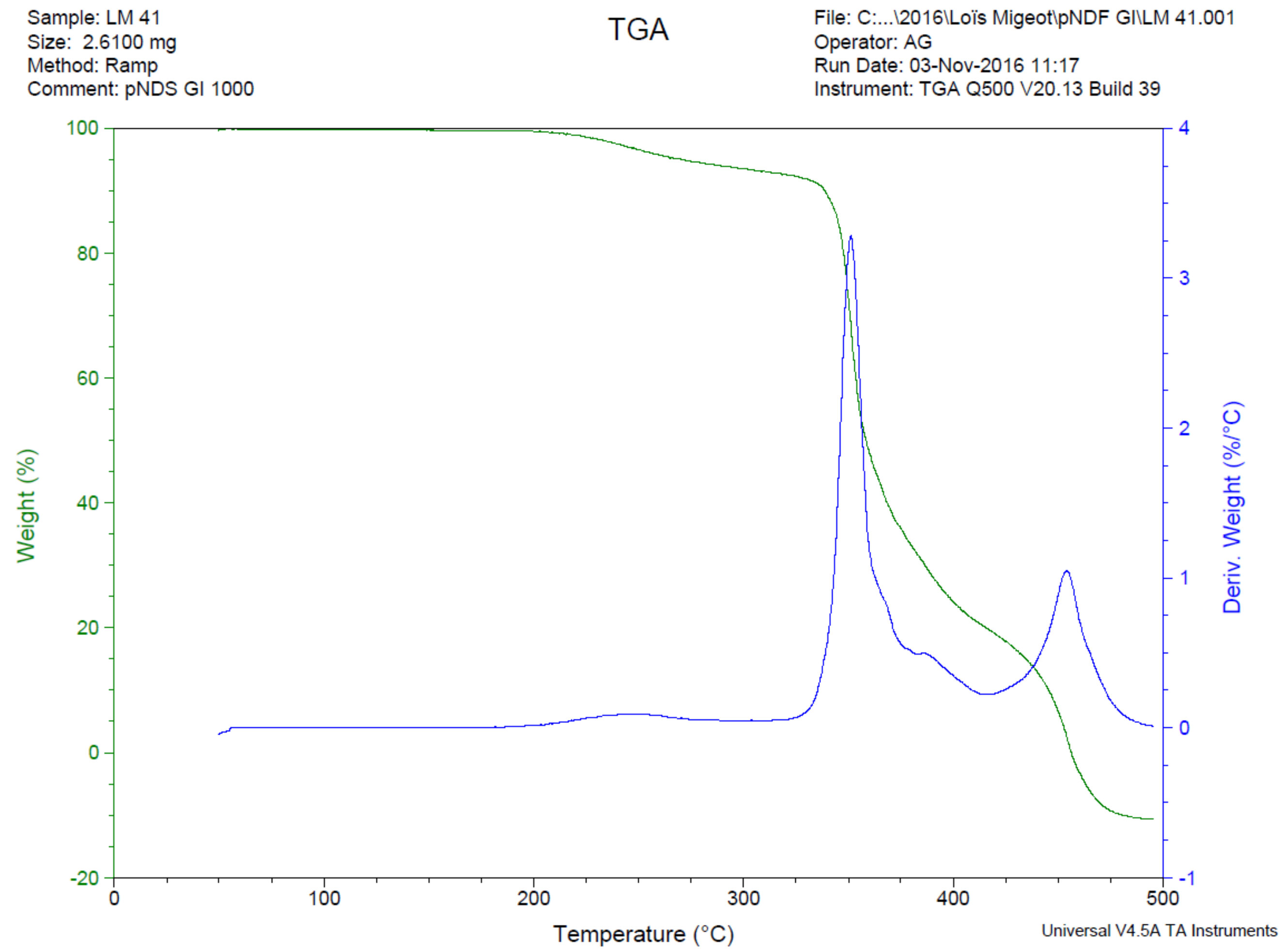

13. DSC analysis of poly(**NDF**)

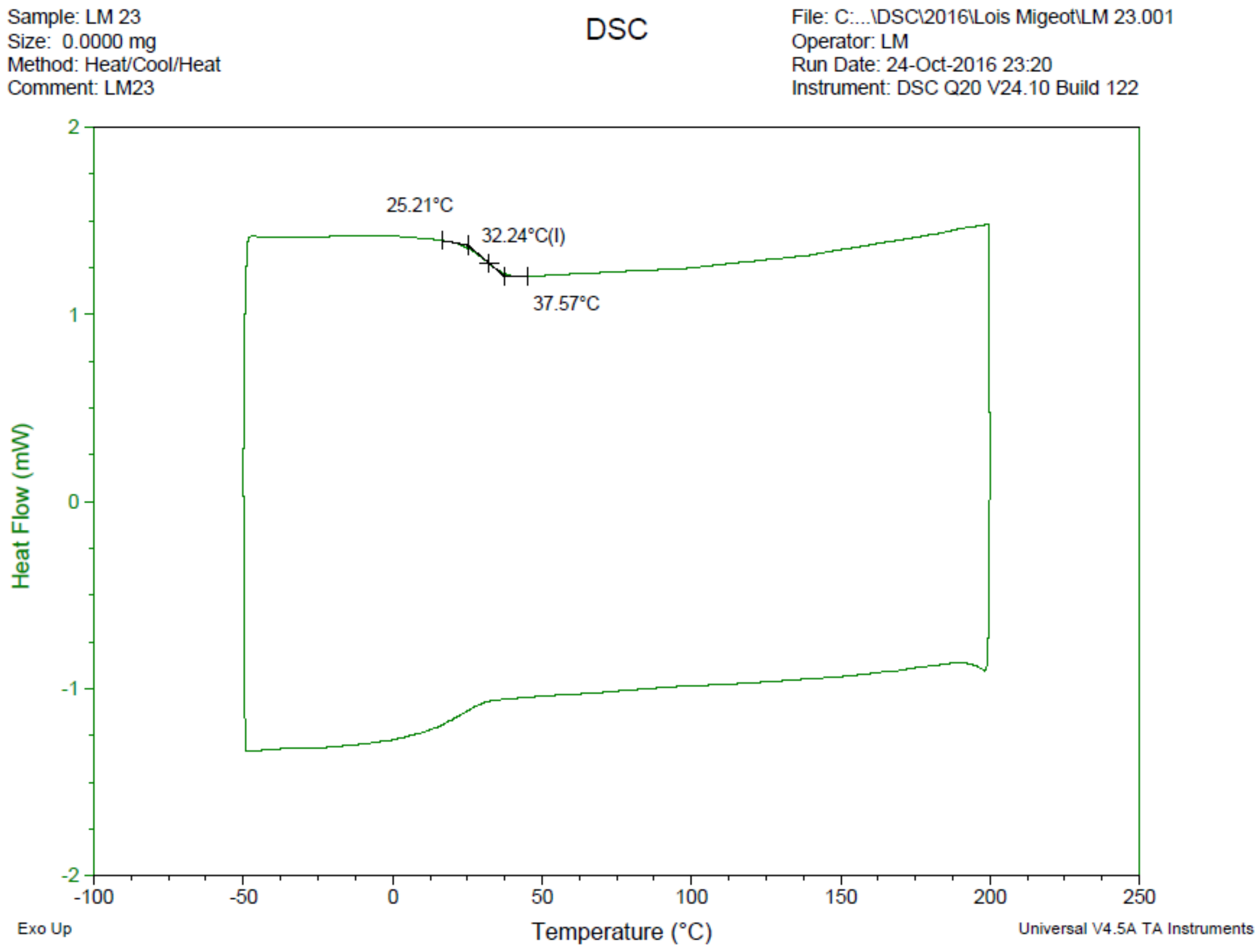

14. DSC analysis of poly(**NDS**)

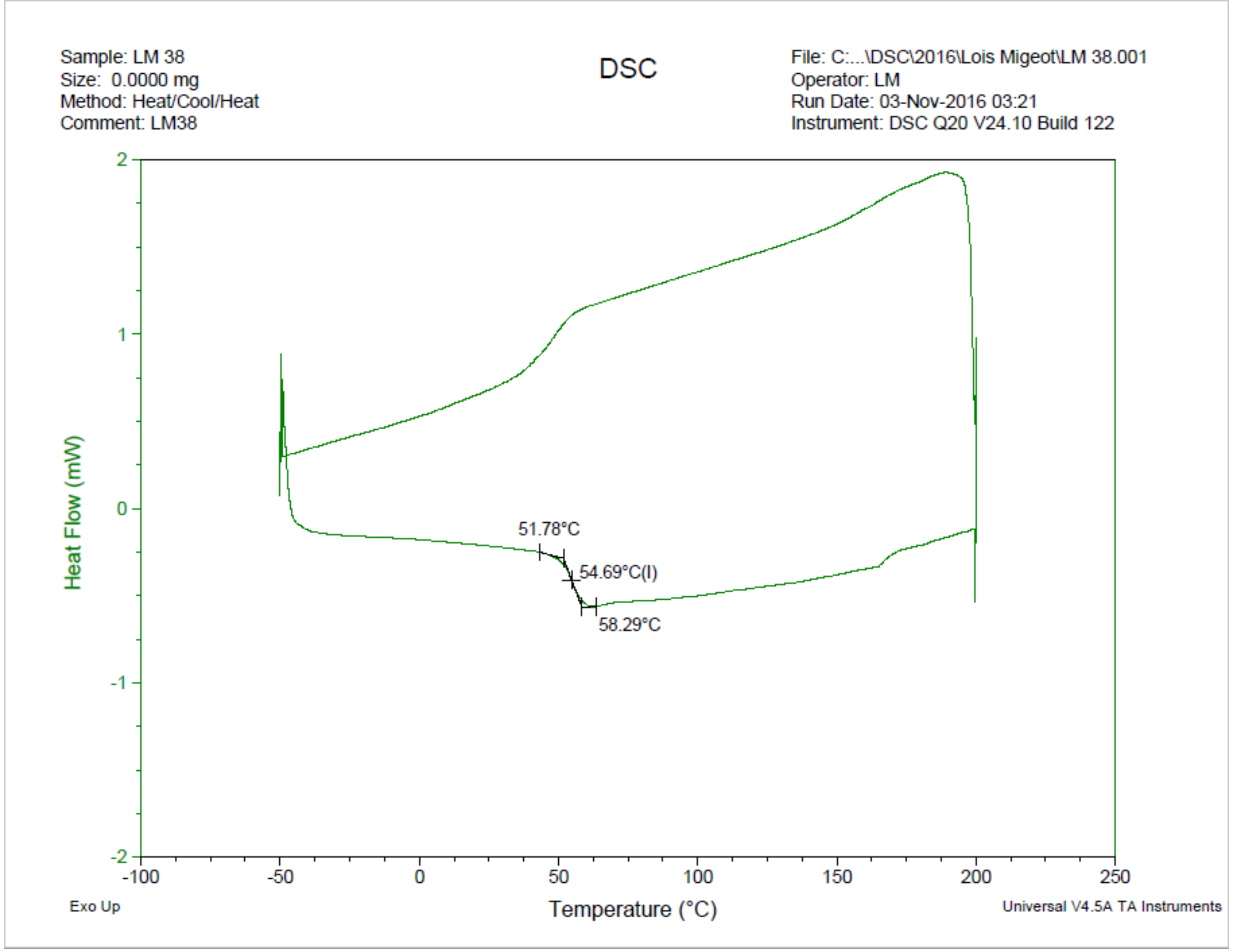

15. GPC analysis of poly(**N**-*co*-**NDF**)

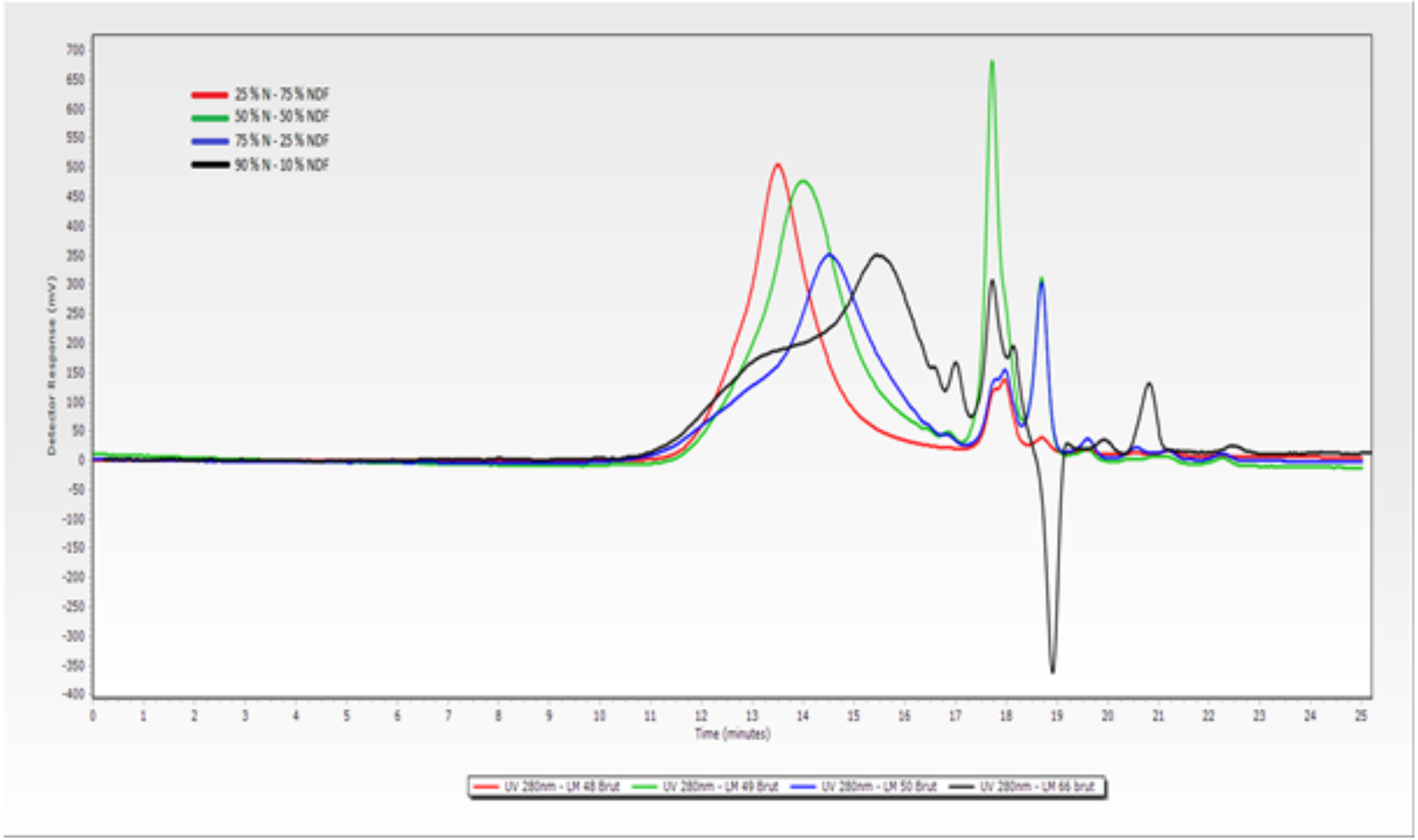

16. GPC analysis of acetone wash method to separate poly(**NDF**) and poly(**N**-*co*-**NDF**)

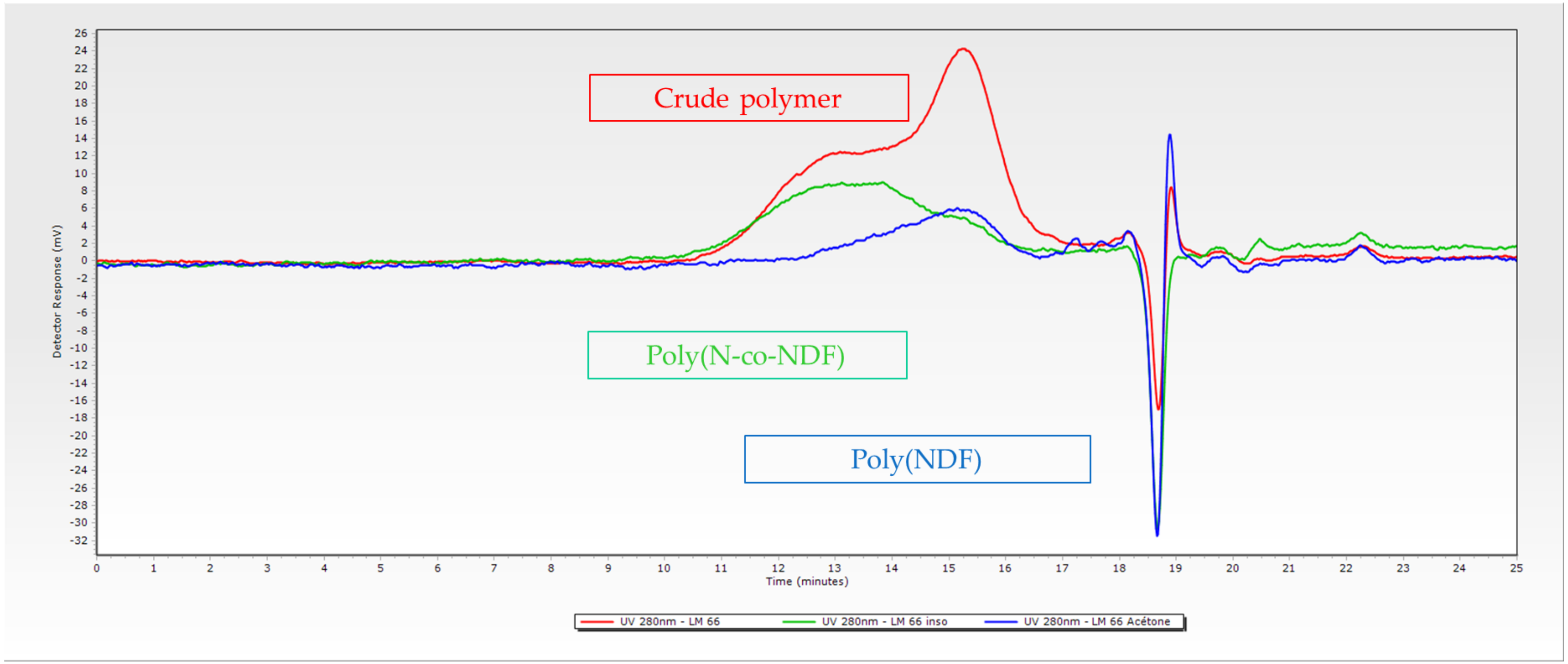

17. ^{1}H NRM spectra of poly(**N**-*co*-**NDF**)

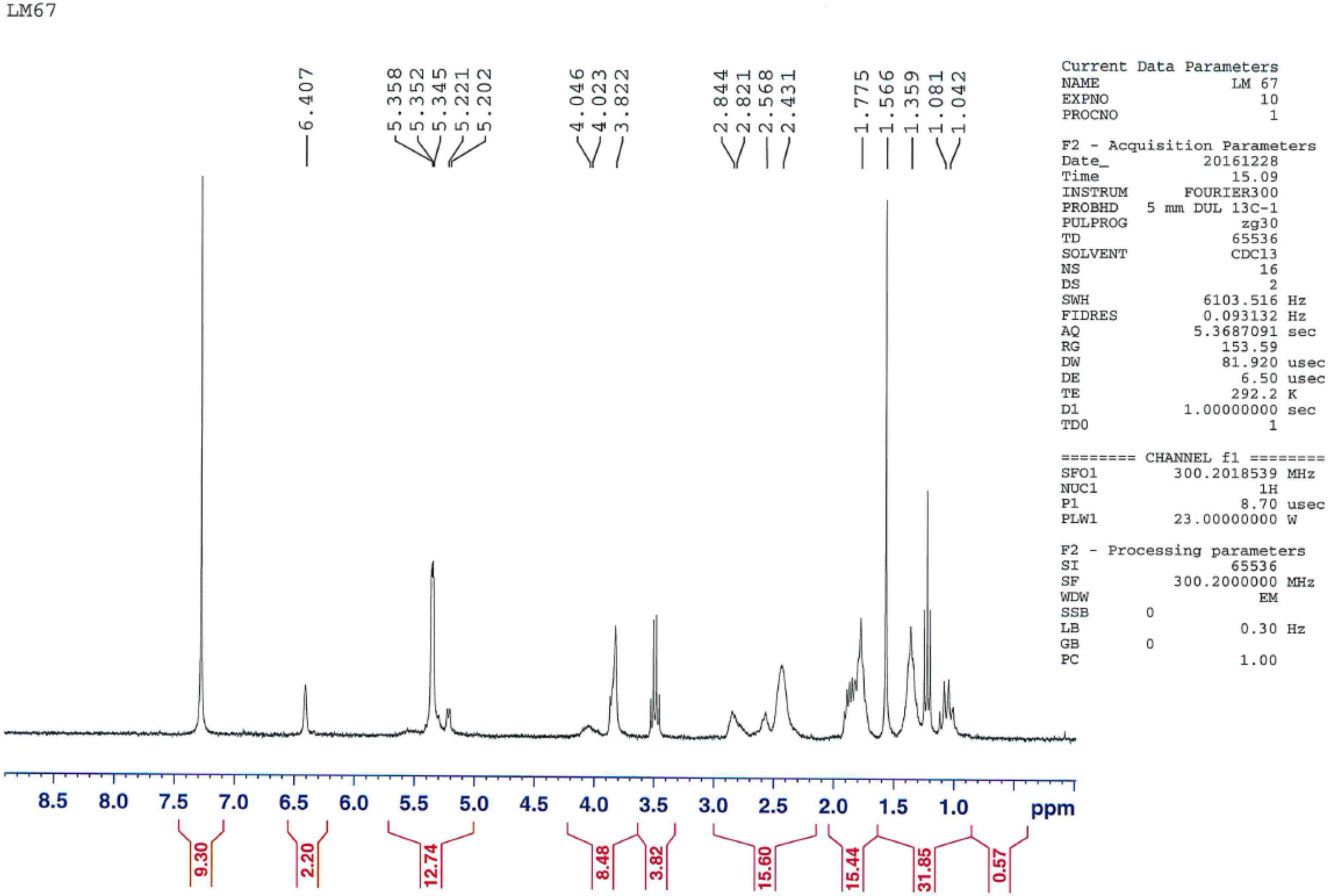


```
Current Data Parameters
NAME                 LM 67
EXPNO                   10
PROCNO                   1

F2 - Acquisition Parameters
Date_             20161228
Time                 15.09
INSTRUM         FOURIER300
PROBHD    5 mm DUL 13C-1
PULPROG               zg30
TD                   65536
SOLVENT              CDCl3
NS                      16
DS                       2
SWH               6103.516 Hz
FIDRES            0.093132 Hz
AQ               5.3687091 sec
RG                  153.59
DW                  81.920 usec
DE                    6.50 usec
TE                   292.2 K
D1              1.00000000 sec
TD0                      1

======== CHANNEL f1 ========
SFO1           300.2018539 MHz
NUC1                    1H
P1                    8.70 usec
PLW1           23.00000000 W

F2 - Processing parameters
SI                   65536
SF             300.2000000 MHz
WDW                     EM
SSB       0
LB                    0.30 Hz
GB        0
PC                    1.00
```

18. GPC analysis of poly(**N**-*co*-**NDS**)

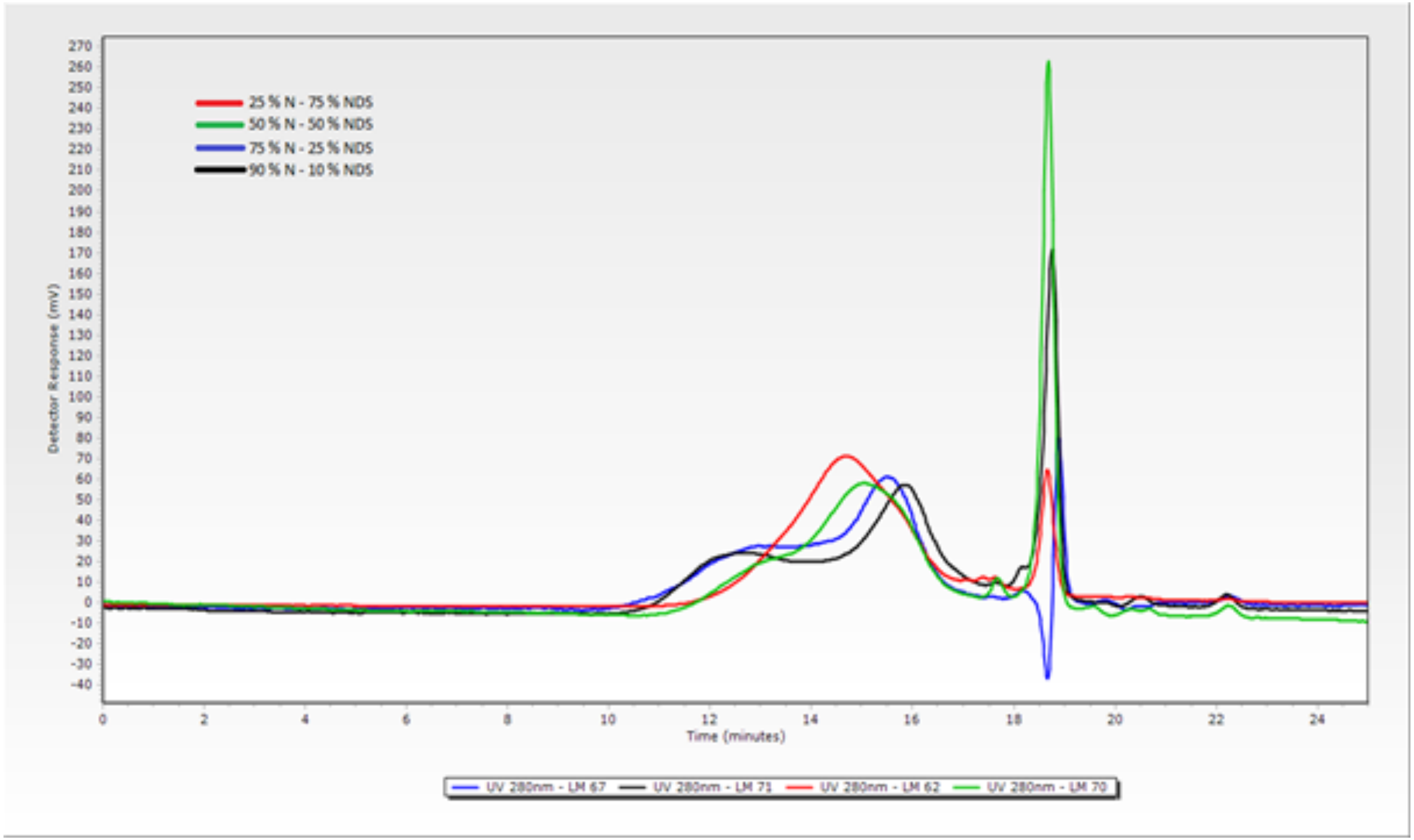

19. GPC analysis of acetone wash method to separate poly(**NDS**) and poly(**N**-*co*-**NDS**)

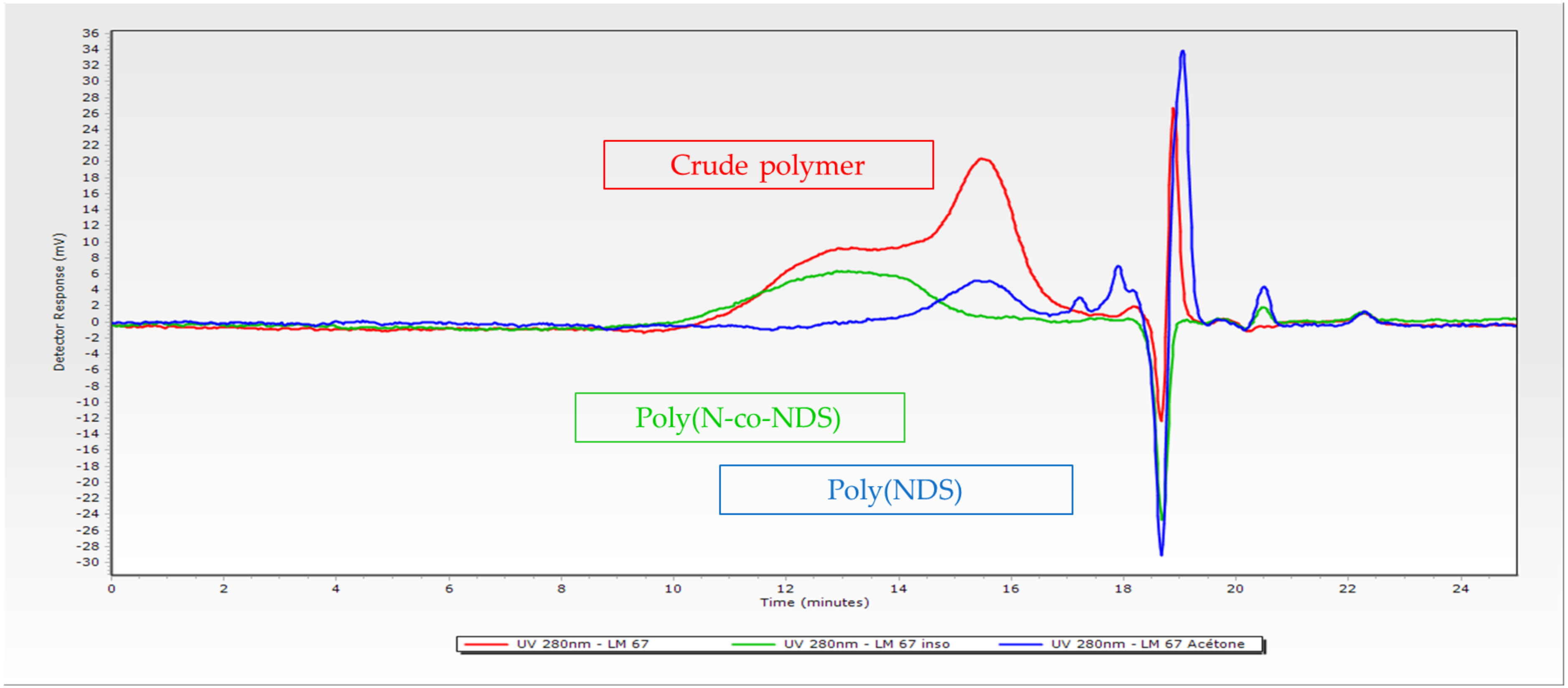

20. ^{1}H NMR spectra of poly(**N**-*co*-**NDS**)

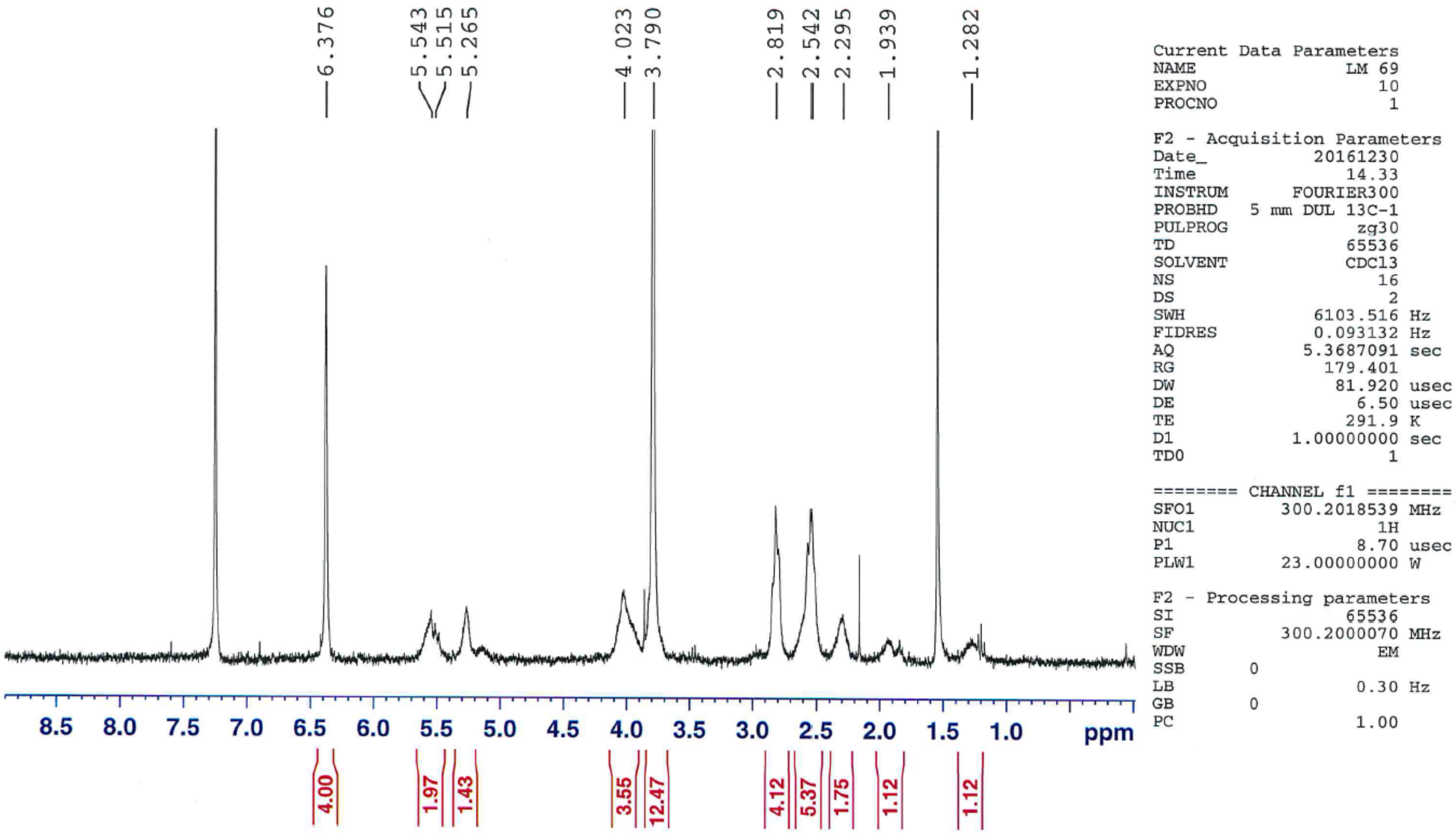

21. TGA analysis of poly(**N**-*co*-**NDF**)

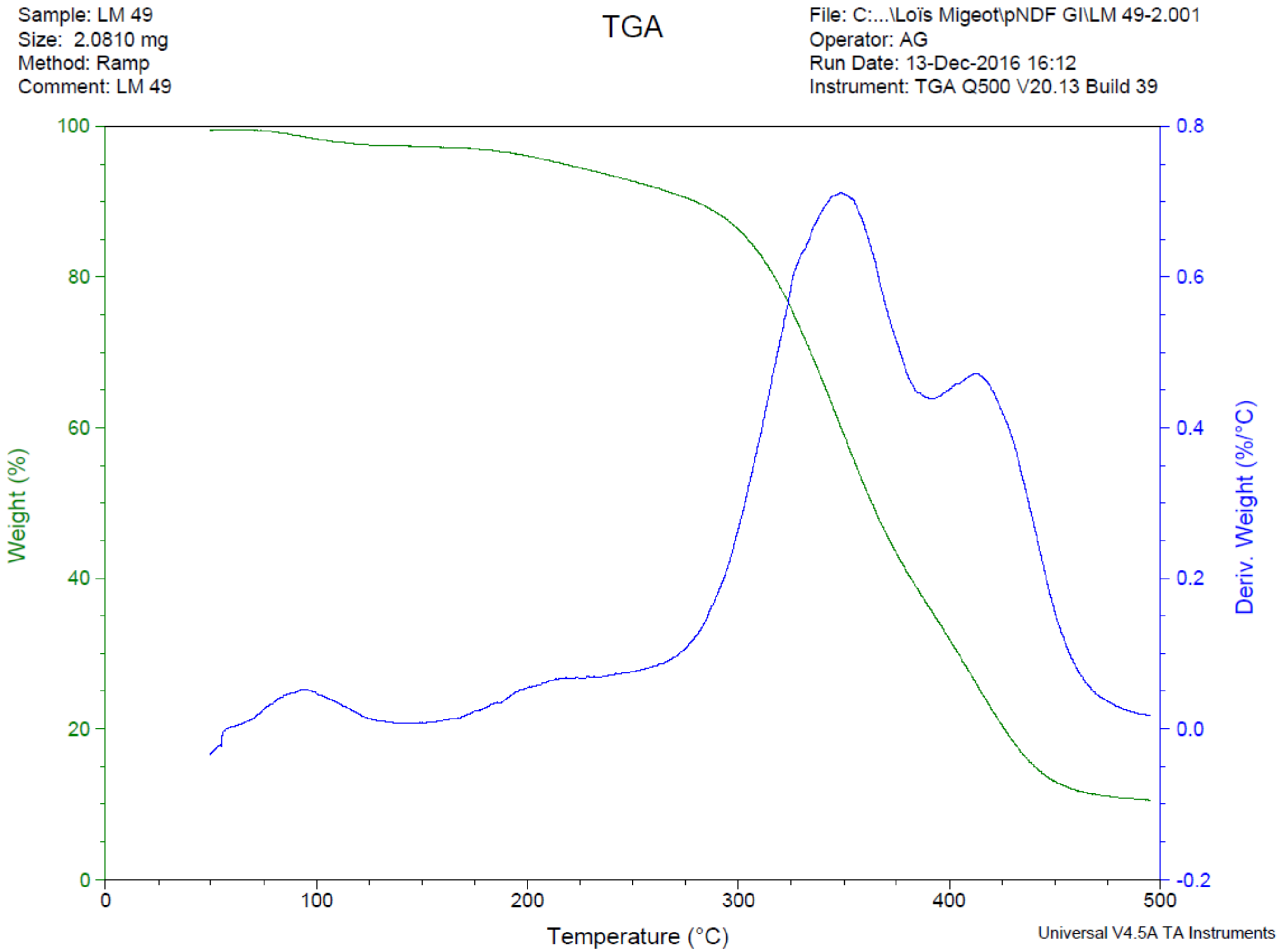

22. TGA analysis of poly(**N**-*co*-**NDS**)

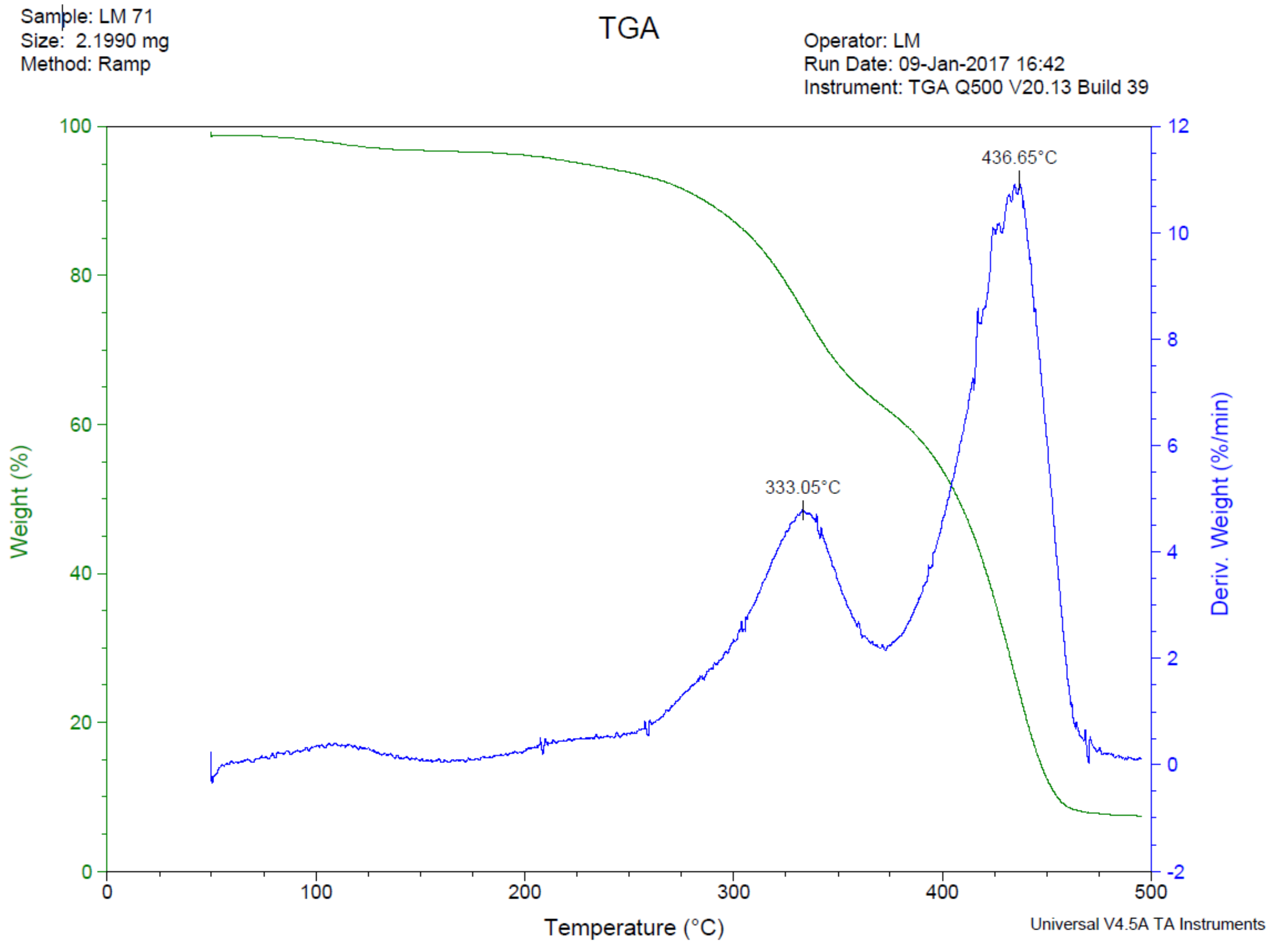

Supplement: Supplementary file 1 [file DataSheet1.PDF]
